# Explore the Anti-Acne Mechanism of Licorice Flavonoids Based on Metabonomics and Microbiome

**DOI:** 10.3389/fphar.2022.832088

**Published:** 2022-02-08

**Authors:** Shi-Fa Ruan, Yi Hu, Wen-Feng Wu, Qun-Qun Du, Zhu-Xian Wang, Ting-Ting Chen, Qun Shen, Li Liu, Cui-Ping Jiang, Hui Li, Yankui Yi, Chun-Yan Shen, Hong-Xia Zhu, Qiang Liu

**Affiliations:** ^1^ School of Traditional Chinese Medicine, Southern Medical University, Guangzhou, China; ^2^ Department of Traditional Chinese Medicine, Guangzhou Red Cross Hospital, Guangzhou, China; ^3^ Integrated Hospital of Traditional Chinese Medicine, Southern Medical University, Guangzhou, China

**Keywords:** acne, licorice flavonoids, metabolites, skin, serum, microbial balance

## Abstract

Acne vulgaris is one of the most common inflammatory dermatoses in dermatological practice and can affect any gender or ethnic group. Although in previous studies, we had found that licorice flavonoids (LCF) play an anti-acne role by inhibiting PI3K-Akt signaling pathways and mitochondrial activity, the mechanism of LCF regulating skin metabolism, serum metabolism and skin microbes is still unclear. Here, we performed a full spectrum analysis of metabolites in the skin and serum using UHPLC-Triple TOF-MS. The results showed that LCF could treat acne by regulating the metabolic balance of amino acids, lipids and fatty acids in serum and skin. Similarly, we performed Illumina Hiseq sequencing of DNA from the skin microbes using 16S ribosomal DNA identification techniques. The results showed that LCF could treat acne by regulating the skin microbes to interfere with acne and make the microecology close to the normal skin state of rats. In summary, this study confirmed the anti-acne mechanism of LCF, namely by regulating metabolic balance and microbial balance. Therefore, this discovery will provide theoretical guidance for the preparation development and clinical application of the drug.

**GRAPHICAL ABSTRACT:**
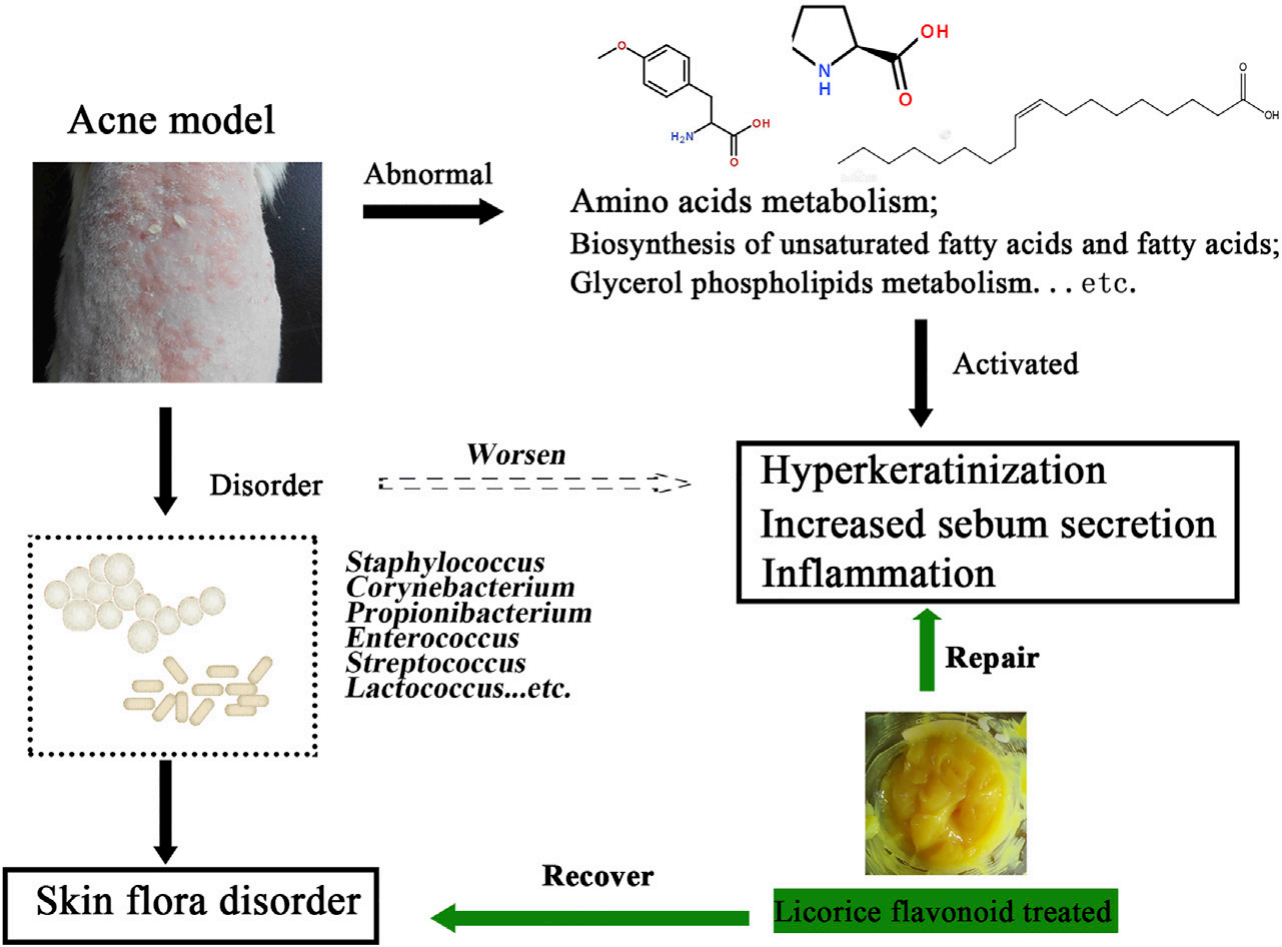


## 1 Introduction

Acne vulgaris is a common chronic inflammatory disease of the skin that causes inflammation or non-inflammatory lesions by affecting the skin’s follicular sebaceous glands (Bershad, 2001; Chen et al., 2021; Zhu et al., 2021). Zaenglein et al. classify acne into three groups, including open acne (blackhead), closed acne (whitehead), and inflammatory lesions such as nodules, pustules, and papules (Zaenglein et al., 2016). Currently, acne is treated primarily with topical therapies (e.g., retinoids, azelaic acid, and fixed combination clindamycin phosphate and benzoyl peroxide gel and tretinoin gel microsphere) and systemic therapies (e.g., antibiotics, oral isotretinoin and anti-androgenic drugs only for women) (Kosmadaki and Katsambas, 2017; Kaminska, 2020; Liu et al., 2020). However, there are few reports on the use of natural products to treat acne.

The natural product licorice flavonoids (LCF) extracted from *glycyrrhiza* is a promising drug for treating acne. Studies had shown that LCF play an active role in regulating lipid biosynthesis and metabolism (Nakagawa et al., 2004). In addition, there are related literature reports that licorice LCF can inhibit the growth of *Cutibacterium acnes*, *Candida albicans*, and *Staphylococcus aureus* (Dai et al., 2013; Kim et al., 2013). However, unfortunately, direct evidence of LCF regulation of skin metabolism, serum metabolism and anti-acne of the skin microbes is still lacking.

Metabolomics is the study of small-molecule metabolites. For skin metabolomics studies, the mechanism of skin response to irritation is elucidated primarily by detecting changes in cellular metabolites (Wu et al., 2014). For example, Murota et al. (2018) confirmed that the glucose concentration in atopic dermatitis (AD) sweat increased with the severity of the disease and the increase of the skin phenotype, suggesting that the increase of blood glucose affected the skin homeostasis. Similarly, serum metabonomics has been widely used in the screening of biomarkers of skin diseases, including psoriasis, and skin cancer etc. For example, Ottas et al. (2017) used both targeted and non-targeted metabolomics methods to analyze the serum of patients with atopic dermatitis, and found that the most significant metabolite changes were associated with phosphatidylcholine, carnitine acyl and its ratio, and fibrinogen A alpha cleavages. Humans and their microbiota develop a variety of innate immune responses to protect the body from infection. Studies have shown that the skin microbiome was in constant and close contact with epithelial cells, regulating intrinsic and acquired immune cell function (Schommer and Gallo, 2013). For example, Zhan Gao et al. (2008) found a microbial ecological imbalance in psoriatic skin lesions, no decrease in the proportion of skin *C. acnes*, and a significantly higher proportion of *streptococcus* than healthy control skin.

Currently, few studies have investigated the direct association between biota and metabolites in LCF anti-acne. The link between skin metabolism, serum metabolism, and skin microbes needs to be explored more deeply. Therefore, in this study, we applied 16SrDNA sequencing and non-targeted metabolomics to determine that the anti-acne mechanism of LCF is by regulating metabolic balance and microbial balance. This work provides a unique perspective to explore the new pathogenic mechanism of LCF in the treatment of acne, which will provide effective theoretical guidance for the development and clinical application of such drugs.

## 2 Materials and Methods

### 2.1 Licorice Flavonoids and Gel Preparation

The licorice (*Glycyrrhiza glabra* L.) (Kangmei Pharmaceutical Co., Ltd., Guangdong, China) was authenticated by Professor XingXing Chan from the College of Chinese Traditional Medicine, Southern Medical University (Guangzhou, China). The herbal materials used in our study satisfy the quality requirement of the Chinese Pharmacopoeia 2020. The extraction process of licorice flavonoids (LCF) and the quantitative analysis of the herbal extracts used are consistent with our previous reports (Ruan et al., 2020).

### 2.2 Analysis of Licorice Flavonoids by UPLC-ESI-Orbitrap-MS

The LCF was reconstituted with methanol and injected on a UPLC-MS instrument. Separation was performed using the Vanquish UHPLC (Thermo Scientific, Waltham, MA, United States) equipped with a UPLC Hypersil Goldanalytical column (2.1 mm × 100 mm, ID1.9 μm) (Thermo Scientific, Waltham, MA, United States). The extractive was eluted using buffer B (.01% formic acid (FA)-acetonitrile) and buffer A (.01% FA), at a flow rate of 200 μl/min. The gradient is as followed: 0–3 min for 100% buffer A, 2–18 min buffer A from 100 to 0% and hold 6 min, then in 24–30 min buffer A back to 100%. The MS was performed on an Orbitrap Fusion system (Thermo Scientific, Waltham, MA, United States) operating in an ESI in both positive and negative modes (positive ion 3,500 V negative ion 2,500 V). Orbitrap resolution was 60,000 with a scan range of 100–1,000 (m/z). The nebulization gas was set to 40 L/h at a temperature of 350°C, and the Curtain gas was set to 10 L/h. For the MS2, cycle time data dependent mode (DDA) was used to acquire data. Data processing and analysis method reference Supplementary Material.

### 2.3 Animals and Treatment

Animal testing mentioned in this research was conducted at the animal facilities of the Animal Ethics Committee of SMU which is consistent with references in the Guide for the Care and Use of Laboratory Animals of China. Studies were carried out on Sprague-Dawley (SD) male rats (6–7 weeks, 180–200 g, male) obtained from the Southern Medical University (SMU) Experimental Animal Center (Guangzhou, China, quality certificate number: SCXK (Yue) 20160041). The modeling method is basically consistent with our previous reports (Ruan et al., 2020). In brief, the rats were divided into four groups, eight in each group, namely, the control group (CTR), the model group (MDL), the licorice flavonoids gel group (LCF), and the negative control group (excluding drug gel) group (NGC). Rats were first anesthetized, their back skin depilated, then applied oleic acid (Aladdin Biochemical Technology Co., Ltd., Shanghai, China) to their back skin once a day, and started dermal injection of acne *C. acnes* (1.8 × 109 CFU/ml) after 7 (Microbial Culture Collection Center, Guangdong, China). After 14 days of modeling, apply the gel to the back skin for 14 days. Rat skin specimens of CTR, MDL, LCF, NGC were taken before being sacrificed.

### 2.4 Western Blot and Serum Analysis

Aliquots of protein samples (eight rats of CTR, MDL and LCF) were used for western blot analysis. The skin was washed with phosphate buffer saline (×1) solution and shredded with tissue scissors, then centrifuged at 13,000 rpm for 10 min, and the supernatant was discarded. The lysis buffer (Beyotime Biotechnology, Shanghai, China) was added and the tissue was disrupted by tissue grinder (Servicebio KZ-II, Wuhan, China). Next, the tissues were lysed at 4°C for 30 min, centrifuged at 16,000 g for 10 min, and the supernatant was collected as a total protein solution. The protein concentration was determined with a BCA protein Quantification kit (Thermo Fisher Scientific, United States). Bands were visualized using the Bio-Rad ChemiDoc XRS digital documentation system (Bio-Rad, United States). The intensity of the bands was normalized to β-actin and quantified using ImageJ version 1.45 software.

Additionally, concentrations of proinflammatory cytokines, including interleukin-8 (IL-8), tumor necrosis factor-α (TNF-α), in serum were quantified utilizing enzyme-linked immunosorbent assay kits, under the instructions of the producer (Nanjing jiancheng bioengineering institute, Nanjing, China).

### 2.5 Skin and Serum Metabolites

#### 2.5.1 Preparation of Skin and Serum Metabolites

The skin samples were weighed and placed in a mortar and grounded with liquid nitrogen. It was then milled 50/50 (v/v) in methanol/water for 15 min, the suspension was transferred to an EP tube and centrifuged at 12,000 rpm for 10 min. Next, the supernatant was concentrated in vacuo and reconstituted by adding 200 μl of methanol. It was then centrifuged for 10 min, and the supernatant was collected. Serum sample of 200 μl was placed in a 1.5 ml centrifuge tube and was ultrasonically extracted with 600 μl of methanol for 30 min, and then centrifuged at 12,000 rpm for 10 min. About 600 μl of the supernatant was vacuum dried and reconstituted by adding 200 μl of methanol. Next, it was centrifuged for 10 min, and the supernatant was collected. Quality control (QC) sample preparation method reference Supplementary Material.

#### 2.5.2 Ultra-Performance Liquid Chromatography Combined With Electrospray Ionization Quadrupole Time-of-Flight Mass Spectrometry (UPLC-ESI-QTOF-MS) Analysis

The 10 μl aliquot was injected into a Nexera UHPLC LC-30A (SHIMADZU, Japan) separation system equipped with Waters HSS T3 column (150 mm × 3 mm, 1.8 µm, Waters, United States). The metabolites were eluted using a 35 min gradient of acetonitrile starting from 95% to 5% over 33 min and gradually back to 95% in the last 2 min at a flow rate of 200 μl/min, and buffer A was 0.1% acetic acid. Column temperature and sample chamber temperature were 35 and 4°C respectively. The MS was performed on a TripleTOF5600+ system (AB SCIEX™, United States) operating in an ESI in both positive and negative modes (positive voltage 5,000 V, negative voltage 4,500 V). Nitrogen was used as nebulization and cone gas. The nebulization gas was set to 80 L/h at a temperature of 600°C, and the Curtain gas was set to 30 L/h. Data between m/z 100 to 1,500 were recorded in the centroid mode. For the MS2, Using Data Independent Acquisition (DIA) scan mode to acquire production in 50–1,000 Da m/z range. Declustering potential, Collision Energy and cone voltage were 60, 35, and 15 V, respectively. In the DIA mode, the isotope in 5 Da is excluded, and seven candidate ions are monitored per cycle. Data processing and analysis method reference Supplementary Material.

### 2.5.3 Bioinformatics Analysis

MetaboAnalyst 4.0 was used for data processing in metabolomics, including data normalization, chemometrics analysis mapping, metabolite identification, and univariate analysis (Student’s t-test and fold change analysis) and enrichment analysis of Kyoto Encyclopedia of Genes and Genomes (KEGG) pathways. Interquartile range and relative standard deviation >25% in QC samples were used in order to filter that data. To identify and remove variables that are unlikely to be of use when modeling the data. No phenotype information was used in the filtering process, so the result can be used with any downstream analysis. Metabolites intensities were log2-transformed and Pareto scaling and used for further statistical analysis. R software was used to draw heatmaps and volcano plots. A statistical model was developed using Ortho Partial Least Squares Discrimination Analysis (OPLS-DA), to reflect the correlation between the expression level of skin metabolites and the sample class in order to predict the sample class. Screening for biomarkers in metabolites by calculating the Variable Importance for the projection (VIP) value with VIP > 0.8. Univariate statistical test was done using Wilcoxon signed-rank sum test and fold change (FC) analysis to assist in the screening of biomarkers. Metabolites that meet FC ≥ 1.5 and *p* < .05, or VIP > .8 were considered as differential expressed metabolites (DEMs). Conformed to both *p* < .05 and VIP value > .8 were considered to be significantly differentially expressed metabolites (SDEMs).

### 2.6 Skin Microbiota

#### 2.6.1 DNA Extraction, PCR Amplification, and Illumina MiSeq Sequencing

Details of DNA Extraction of skin microbes, PCR Amplification have been reported before by our team, and detailed experimental operations could be obtained in it (Tang et al., 2018). PE amplicon libraries were established, and sequencing was conducted utilizing the Illumina MiSeq platform at Majorbio Bio-Pharm Technology Co. Ltd., Shanghai, China. Raw fastq files were demultiplexed and quality-filtered utilizing FLASH and Trimmomatic.

### 2.6.2 Bioinformatics Analysis

The operational taxonomic units (OTUs) that reached a 97% nucleotide similarity level were subjected to alpha-diversity analyses using mothur software (Jeremy et al., 2003). Beta-diversity measurements were calculated as previously described (Oh et al., 2014), and principal coordinate analyses (PCoA) on the basis of OTU abundance and distance were verified. R package was utilized for the visualization of bacterial community classification and distribution. For linear discriminant analysis effect size (LEfSe) (Segata et al., 2011), biological relevance and statistical significance were taken into account, and it was used to identify taxa at skin sites that were either enriched or depleted specifically among the three groups. Microbial functions were forecasted utilizing phylogenetic investigation of communities by reconstruction of unobserved state (PICRUSt) (Wang et al., 2014). The predicted genes and their functions are in line with the KEGG database and compared to the STAMP software (http://kiwi.cs.dal.ca/Software/STAMP) (Dreno et al., 2017).

### 2.7 Statistical Analysis

Statistical analysis was performed using SPSS 21.0 software. The categorical data were represented by $\bar{X}\pm S$ and analyzed by one-way ANOVA. The ranked data were analyzed using the Kruskal-Wallis H test. The difference was statistically significant at *p* < .05 or *p* < .01.

## 3 Results

### 3.1 The UPLC-Orbitrap-MS Analysis of the Licorice Flavonoids

**FIGURE 1:**
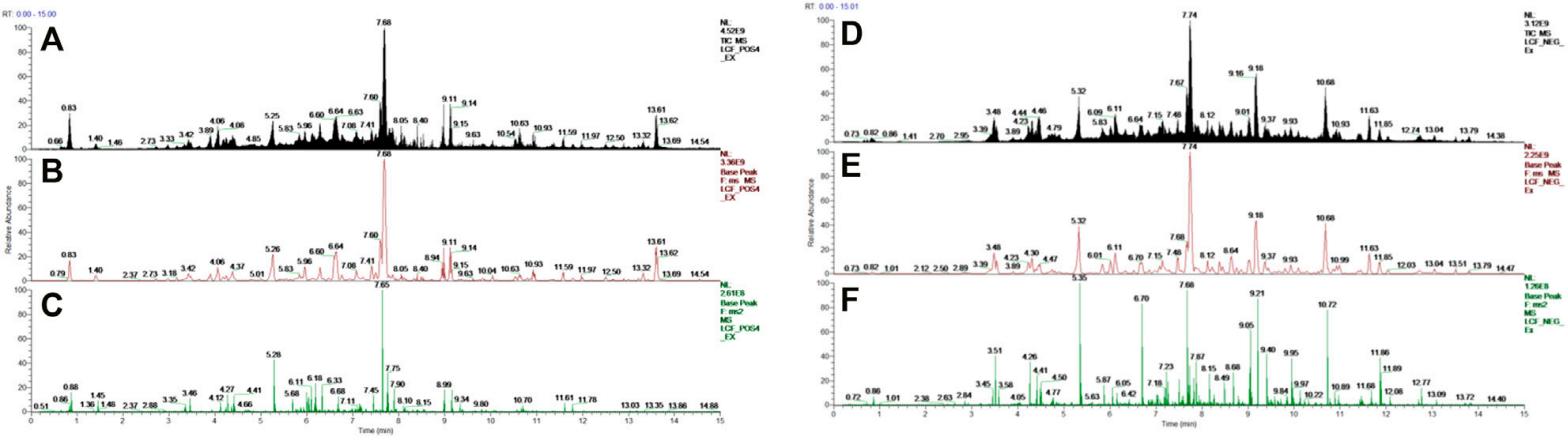
UHPLC-Orbitrap-MS spectrometry of LCF. **(A,D)** Total ion current chromatograms; **(B,E)** base peak of MS; **(C,F)** base peak of MS2.

UHPLC-Orbitrap-MS spectrometry was used to characterize the chemical LCF composition. The total ion current chromatograms of the LCF are shown in Figure 1. Seventy-four major compounds were identified and quantified in relative contents, including amounts of flavonoid like Licochalcone C (LCC) (20.4%), Neobavaisoflavone (13.8%), Licochalcone A (LCA) (12.9%), Diammonium glycyrrhizinate (5.5%), Licoflavone A (4.4%), Liguiritigenin (4.3%), Sesamolin (2.7%), Dimefuron (2.6%), Kanzonol C (2.1%), Isoliquiritigenin (1.9%), Glycyrrhizic acid (.8%), Liquiritigenin (.7%), Glabrolide (0.5%), and a few of organic acids and alkaloids like Ursolic acid (1.4%), Azelaic acid, Proline, and Berberine. Detailed compounds of LCF are shown in Supplementary Table S1.

### 3.2 Pharmacodynamic Analysis of Licorice Flavonoids Gel Anti-Acne

**FIGURE 2:**
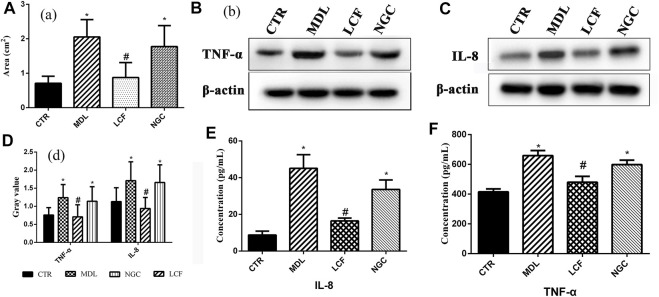
Pharmacodynamic analysis of licorice flavonoid gel anti-acne; Histological analysis: **(A)** Area of the stratified squamous epithelium; Area of the stratified squamous epithelium; Western blot of skin protein: **(B)** TNF-α, **(C)** IL-8, **(D)** Gray values of TNF-α and IL-8; **(E,F)** Serum concentration of TNF-α and IL-8.

By counting the area of the stratified squamous epithelium of each skin sample, the degree of lesions of the acne and the therapeutic effect of the drug was quantified. The result was presented in Figure 2A. Compared with CTR, the area of the epidermis in the MDL increased significantly, with a significant difference (*p* < .05), indicating that the modeling was effective. The epidermal area in NGC was still significant (*p* < .05), indicating that the gel matrix could not improve the hyperkeratosis of acne skin. Compared with MDL, the area of the epidermis in LCF was significantly reduced, and the difference was significant (*p* < .05), indicating that LCF can improve the pathological condition of acne.

TNF-α and IL-8 are important cytokines in the inflammatory response of acne (Lee et al., 2017). Compared with the CTR group, the expression levels of TNF-α (Figure 2B) and IL-8 (Figure 2C) in the skin were significantly upregulated in the model group and the blank gel group (*p* < .05). ELISA results showed that serum TNF-α (Figure 2F) and IL-8 (Figure 2E) were also significantly upregulated (*p* < .05) in the MDL group and the NGC group. In the comparison MDL group, the expression levels of TNF-α and IL-8 in both skin and serum were significantly decreased (*p* < .05) after treatment with LCF, indicating that LCF can inhibit the inflammatory response in acne.

### 3.3 Metabolomics Data Preprocessing and QC Samples Assessment

**FIGURE 3:**
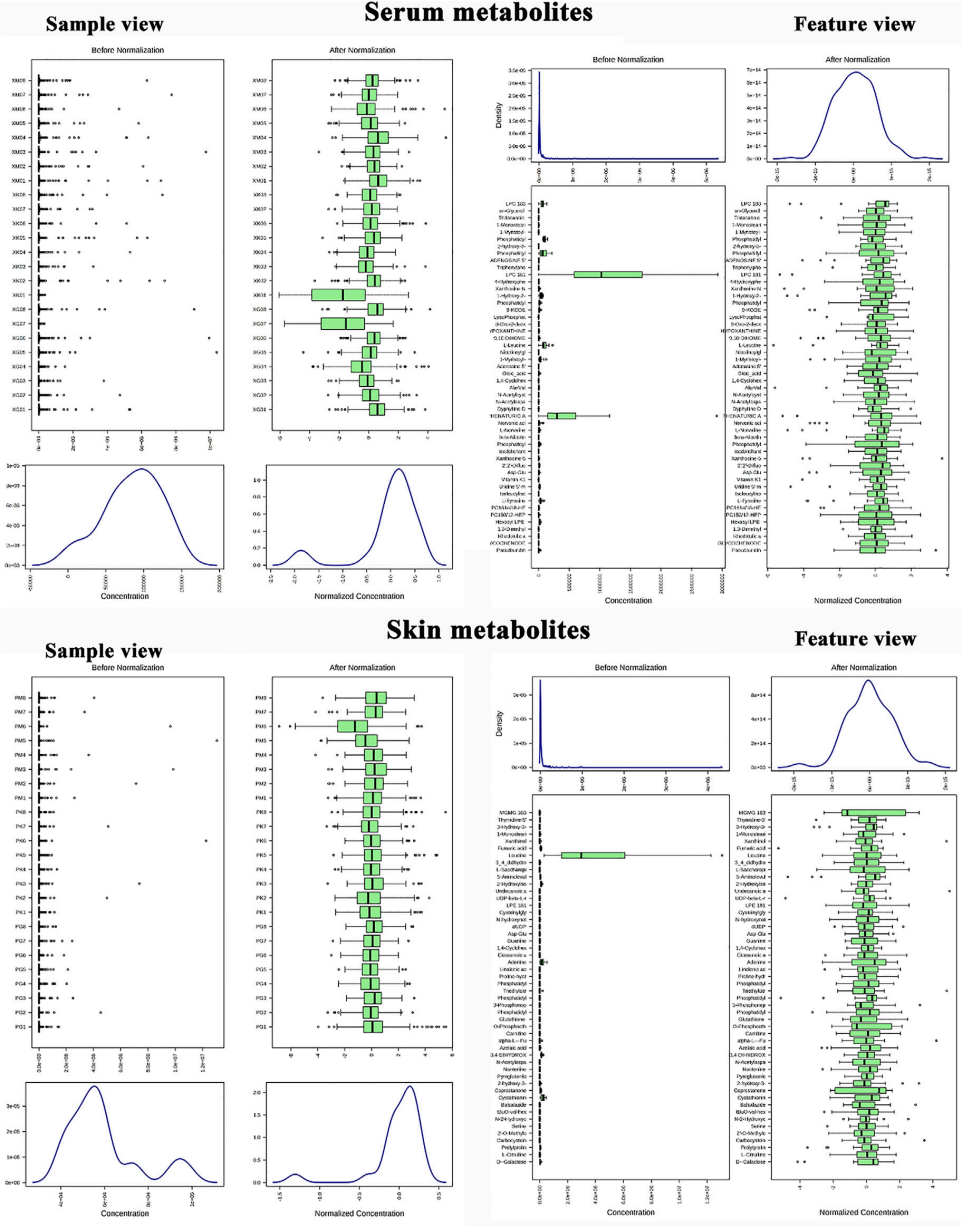
Metabolomics data preprocessing; Skin metabolite data before and after normalization (sample feature view); Serum metabolite data before and after normalization (sample feature view).

**FIGURE 4:**
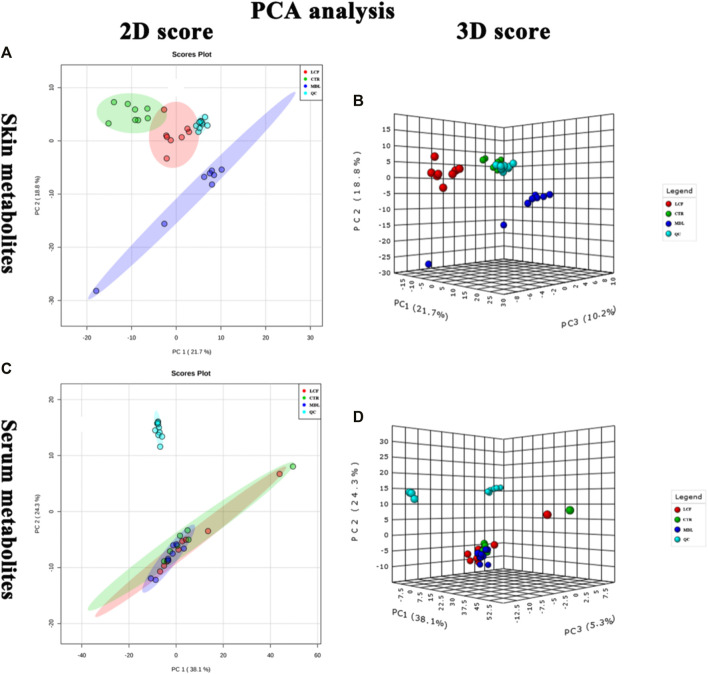
QC samples assessment; **(A)** PCA 2D plot of skin metabolites; **(B)** PCA 3D plot of skin metabolites; **(C)**: **(A)** PCA 2D plot of serum metabolites; **(D): (A)**: PCA 3D plot of serum metabolites.

Metabolite data before and after normalization could be seen in Figure 3. The results of Principal component analysis (PCA) showed that the QC samples were tightly aggregated, indicating that the chromatographic method was reliable, highly reproducible, and the experimental results were stable and reliable. The differential metabolic profile obtained in the sample reflects the biological differences between the samples. PCA result was represented in Figure 4.

### 3.4 Cluster Analysis

**FIGURE 5:**
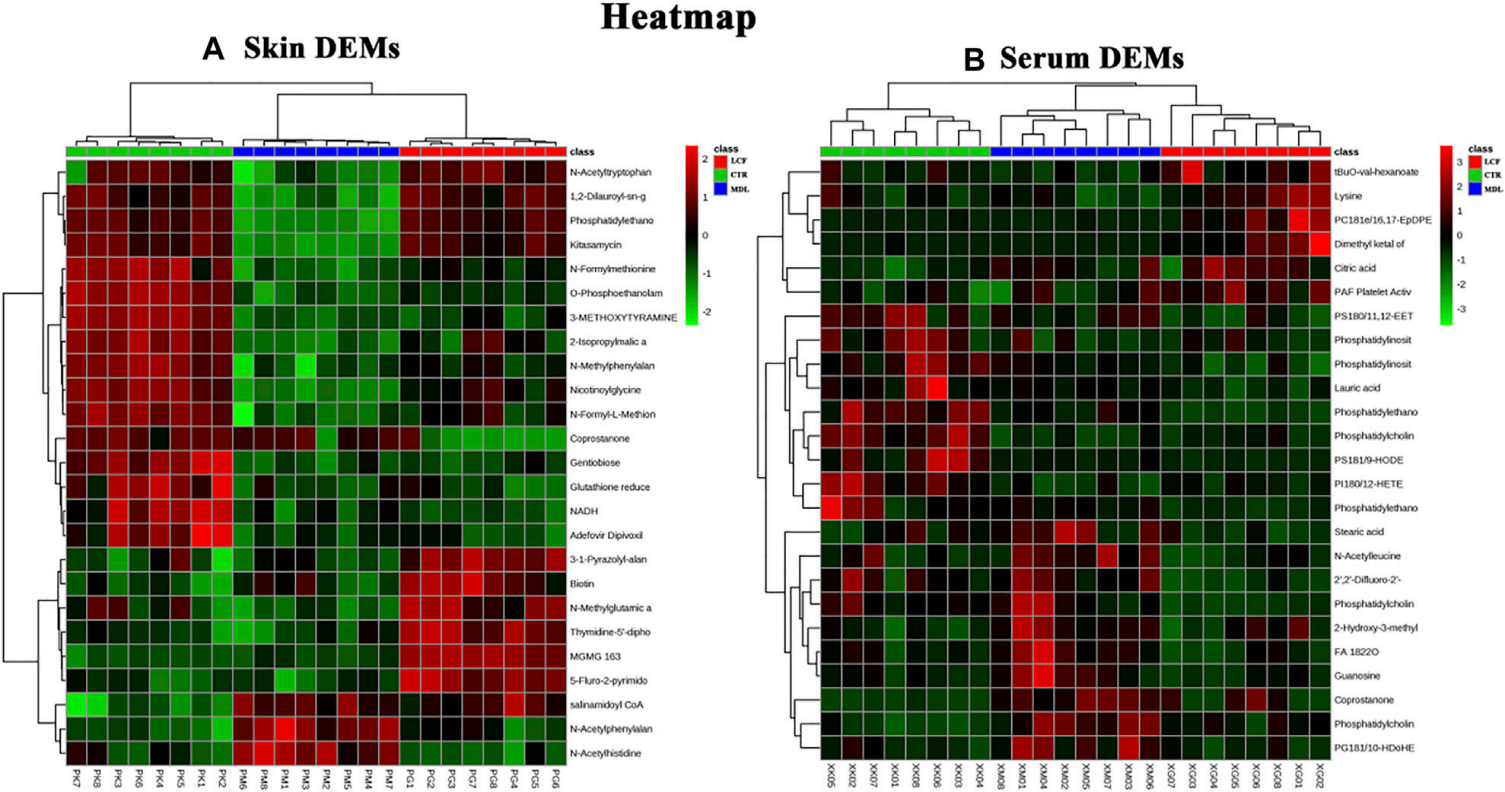
Cluster analysis of DEMs; **(A)** heat map of skin DEMs; **(B)** heat map of serum DEMs Group: LCF (RED), CTR (GREEN), MDL (BLUE). Heat map: upregulation in red, downregulation in green.

The metabolites data were normalized by auto-scale, and the sample distance was calculated using Euclidean. The clustering algorithm was Ward. The top 25 DEMs in the skin and serum metabolites were screened by one-way ANOVA, and post-hoc tests were shown on the heat map, on which, three groups of skin samples (Figure 5A) and serum samples (Figure 5B) can be clustered well, and different metabolites with similar characteristics can be clustered well.

### 3.5 Skin Metabolomics Analysis

#### 3.5.1 Ortho Partial Least Squares Discrimination Analysis

**FIGURE 6:**
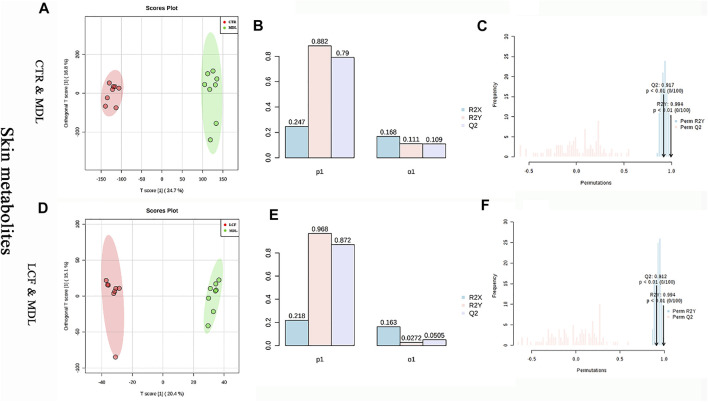
OPLS-DA statistical model between skin metabolites. **(A)** CTR and MDL OPLS-DA plot, **(D)** LCF and MDL OPLS-DA score map, **(B/E)** OPLS-DA parameter test, **(C/F)**: OPLS-DA alignment test.

OPLS-DA was performed on CTR and MDL, LCF and MDL, respectively. CTR and MDL could be completely separated in OPLS-DA, and the samples in each group could be well aggregated (Figure 6A). The statistical model was tested for parameters. The R2Y represented the prediction rate of the group, was 88.2%, and Q2 represents the accuracy of the model prediction was .79, (Figure 6B). To prevent false positives, the statistical model was tested with 100 response alignment tests, after that R2 = .994 and Q2 = .917 (Figure 6C). In MDL and LCF OPLS-DA plot, groups could also be completely separated (Figure 6D), and R2Y and Q2 were .994 and .912 after the alignment test, respectively (Figure 6F). The OPLS-DA statistical model was great with prediction rate and accuracy for skin metabolites.

#### 3.5.2 Analysis of Differential Expressed Metabolites and Significantly Differentially Expressed Metabolites in Skin

Compared with MDL, a total of 126 DEMs, seven of which were SDEMs, were identified in CTR. A total of 95 DEMs, seven of which were SDEMs were identified in LCF. SDEMs were represented in Supplementary Tables S2, S3. Among the three groups of DEMs, 61 DEMs were upregulated (or downregulated) in MDL compared to CTR. After the intervention of LCF, these 61 DEMs were downregulated (or upregulated) compared to MDL, indicating LCF can reverse the metabolic disorder state of rat skin acne. Compared with CTR, leucine was upregulated in MDL (VIP > .8), aspartate and M-tyrosine were significantly upregulated (VIP>1, *p* < .05). Captopril, Phosphatidylethanolamine 16 (PE16) and minobenzoyl-glutamat were significantly downregulated (VIP > 1, *p* < .05).

After administration of LCF, Captopril and PE16, 5-Fluro-2-pyrimidone were significantly upregulated (VIP > 1, *p* < .05) and Aminobenzoyl-glutamat was upregulated (*p* < .05) in LCF compared with MDL. The leucine expression in LCF was significantly lower than that of MDL (VIP = 23.2, *p* = .003), and M-tyrosine was downregulated compared to MDL.

#### 3.5.3 Kyoto Encyclopedia of Genes and Genomes Enrichment Analysis

All DEMs were assigned to the KEGG ID. The KEGG pathway analysis was based on the Over Representation Analysis of the Hypergeometric Test, and the species is *Rattus norvegicus*. All DEMs were enriched into 36 pathways (Supplementary Table S4). Alanine, aspartate, and glutamate metabolism, arginine and proline metabolism, aminoacyl-tRNA biosynthesis, histidine metabolism and beta-alanine metabolism, which were activated in MDL. Aminoacyl-tRNA biosynthesis, valine, leucine and isoleucine biosynthesis, and valine, leucine and isoleucine degradation pathways were inhibited in LCF.

### 3.6 Serum Metabolomics Analysis

#### 3.6.1 Ortho Partial Least Squares Discrimination Analysis

**FIGURE 7:**
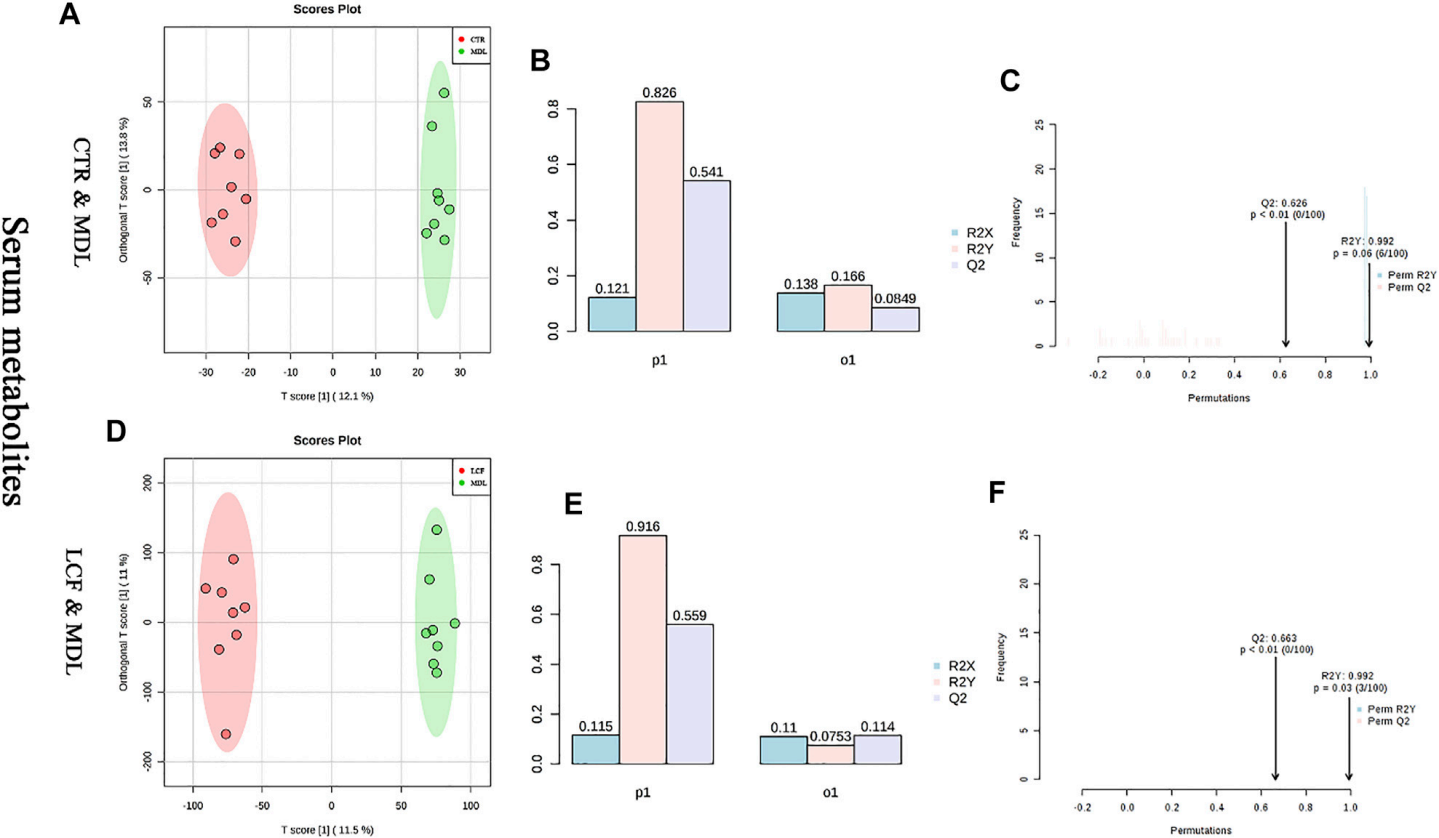
Analysis of OPLS-DA model between serum metabolites; **(A)** CTR vs. MDL OPLS-DA score map, **(D)**: LCF vs. MDL OPLS-DA score map, **(B/E)**: OPLS-DA parameter test, **(C/F)**: OPLS-DA alignment test.

OPLS-DA was performed on CTR and MDL, and LCF and MDL, respectively. In the OPLS-DA plot, either CTR and MDL or MDL and LCF could be differentiated completely (Figures 7A–D). The alignment test showed that R2Y and Q2 were .992, .626, respectively, in CTR and MDL (Figure 7C). In MDL and LCF, R2Y and Q2 were .992 and .663 (Figure 7F). The OPLS-DA statistical model could accurately reflect the separation of two groups of samples and had a good prediction rate.

#### 3.6.2 Analysis of Differential Expressed Metabolites and Significantly Differentially Expressed Metabolites in Serum

Comparing CTR with MDL, a total of 116 DEMs were identified, 12 of which were SDEMs. A total of 102 DEMs were identified by comparing LCF with MDL, 11 of which were SDEMs. SDEMs of serum were represented in Supplementary Tables S5, S6.

In MDL, the level of succinic acid, hydroxybutyric acid, stearic acid (SA), trans-vaccenic acid, phenaturic acid, OA, docosahexaenoic acid, cholic acid, thymidine, and L-phenylalanine was upregulated. Moreover, coprostanone, DL-beta-hydroxybutyric acid, lysophosphatidylethanolamine 18: 1 (LPE18: 1), lysophosphatidylcholine 16 (LPC16), and LPC18: 2 were significantly upregulated (VIP > .8, *p* < .05). Leucine and phenylalanine were downregulated in MDL. In LCF, the serum metabolites that upregulated in MDL were downregulated in LCF. Among them, stearic acid, cholic acid, trans-vaccenic acid, LPC16, OA, LPE18: 1, coprostanone, and thymidine were significantly downregulated (VIP > .8, *p* < .05). The level of leucine and phenylalanine was upregulated in LCF, and the phenylalanine was significant (VIP > .8, *p* < .05). In addition, proline was significantly upregulated in LCF (VIP > .8, *p* < .05) compared to the downregulation of L-Proline in MDL (*p* < .05). Results showed that LCF can restore the inordinate serum metabolites of acne rats.

#### 3.6.3 Kyoto Encyclopedia of Genes and Genomes Enrichment Analysis

Serum DEMs were involved in 28 metabolic pathways, which were listed in Supplementary Table S7. By regulating SDEMs, Phenylalanine metabolism and biosynthesis, Aminoacyl-tRNA biosynthesis, Valine, leucine and isoleucine biosynthesis, Fatty acid elongation in mitochondria, Valine, leucine and isoleucine degradation and Fatty acid metabolism were inhibited in MDL but activated in LCF. Moreover, Biosynthesis of unsaturated fatty acids, Fatty acid biosynthesis, Alanine, aspartate and glutamate metabolism were activated in MDL but restrained in LCF.

### 3.7 Skin Microbiota Analysis

#### 3.7.1 Response of the Skin Microbiota Structure to the Licorice Flavonoids in Acne Rats

**FIGURE 8:**
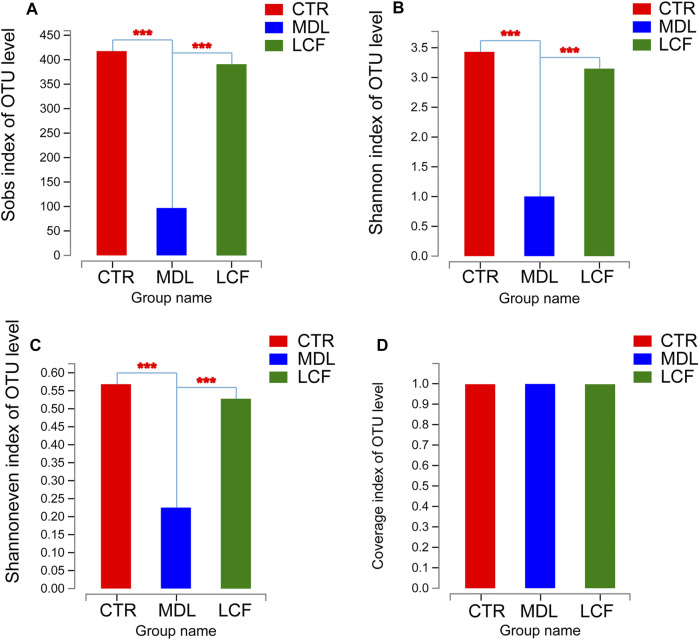
Alpha diversity comparisons of microbial communities of CTR, MDL and LCF. **(A)** The calculated Sobs species richness indices for CTR, MDL and LCF. **(B)** The calculated Shannon species diversity predictions for each group; **(C)** The Shannoneven species evenness indices for the three groups; **(D)** The Good’s coverage for the community in CTR, MDL, and LCF. ***p < .001.

16S rRNA gene sequencing was used to investigate whether LCF had an effect on the structure of skin microbiota in acne rats. Alpha diversity is an ecological measure of how many taxonomic groups are present within each sample and whether the abundance of these groups is evenly distributed (Tang et al., 2018). Compared to CTR, MDL, and LCF samples, alpha diversity was significantly reduced in the MDL, using either the Sobs richness metric (Figure 8A) or the Shannon entropy metric (non-parametric test, all *p* values < .001) (Figure 8B). MDL skin samples were significantly lower in diversity compared to LCF, using the Shannoneven evenness indices (Figure 8C). Good’s coverage beyond 99.7% demonstrated an adequate sequencing depth for all samples.

**FIGURE 9:**
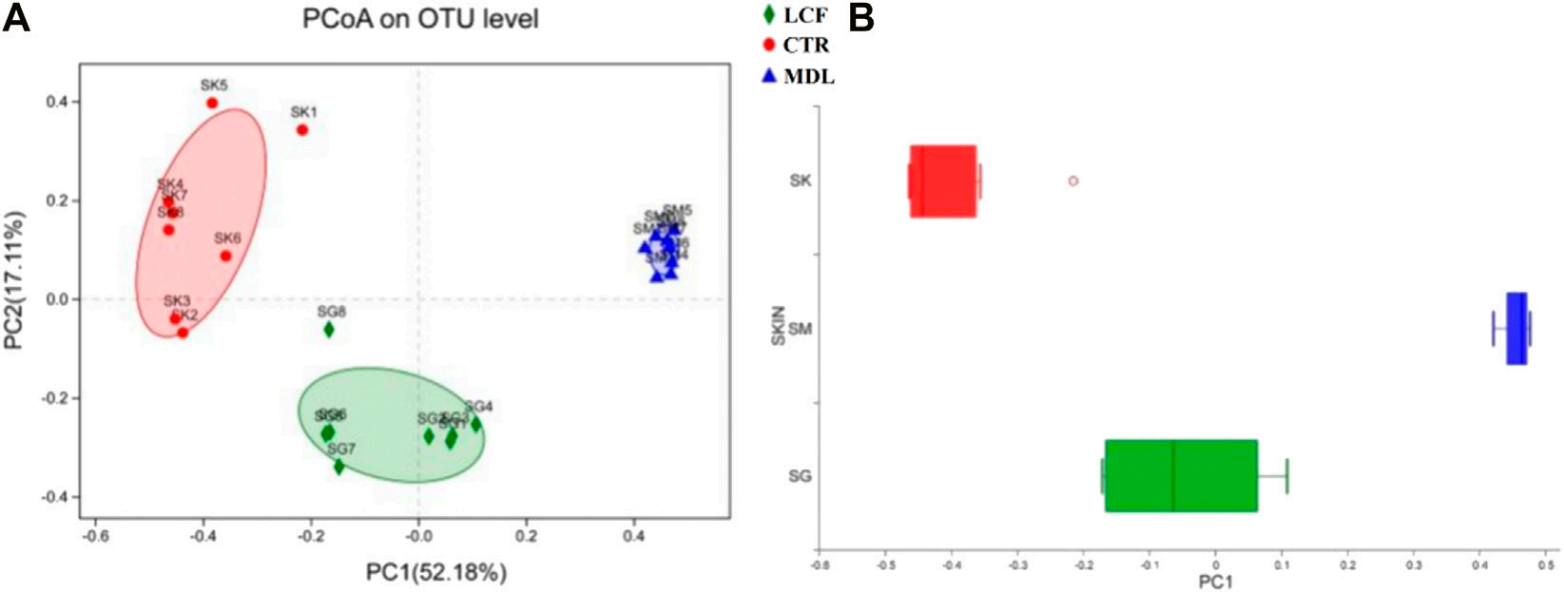
Beta diversity comparisons of microbial communities of CTR, MDL, and LCF. Displayed are **(A)** Primary coordinate analyses of skin microbes samples of abundance UniFrac distances between samples from LCF, MDL, and LCF, and **(B)** Discrete box plot of grouped samples on the PC1 axis of PcoA.

To characterize similarities and differences in the composition of microbial communities, we calculated the weighted UniFrac distances of beta diversity. The community composition of skin samples was significantly different among CTR, MDL and LCF in PCoA (Figure 9A). The LCF sample’s community composition was closer to CTR than MDL in the discrete box plot (Figure 9B).

#### 3.7.2 Key Phylotypes of Skin Microbiota Modulated by the Licorice Flavonoids

**FIGURE 10:**
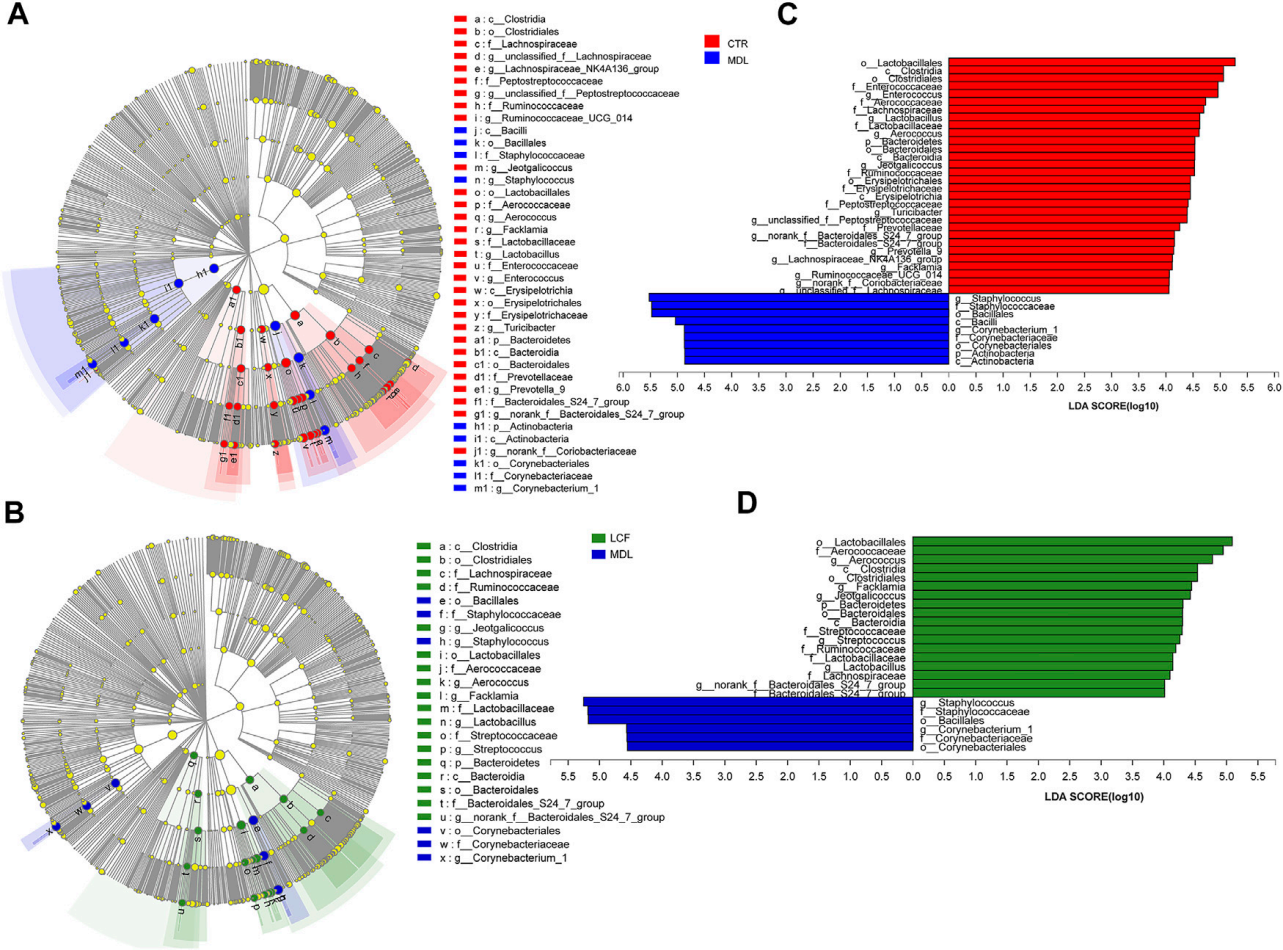
Key bacterial alterations in response to the acne and LCF treatments. **(A)** Cladogram generated by the LEfSe analysis (LDA = 4) showing enriched or depleted taxa in rat skin samples from the CTR (red) and MDL (blue) groups. **(B)** Cladogram generated by the LEfSe analysis (LDA = 4) showing enriched or depleted taxa in rat skin samples from the LCF (green) and MDL (blue) groups. **(C)** LDA scores of enriched taxa are shown in **(A)**. **(D)** LDA scores of enriched taxa are shown in **(B)**. n=8 rats per group.

To further define the acne-associated microbiota in MDL and LCF, LEfSe (Figure 10) was used to identify taxa at skin site that were either enriched or depleted specifically in rat samples based on the linear discriminant analysis (LDA) values of 4 (LEfSe *p* values < .05 for all taxa listed as enriched or depleted). At the phylum level, the MDL samples were enriched for Actinobacteria, LCF samples were enriched for Bacteroidetes. At the genus level, the MDL samples were enriched for four common pathogens *Staphylococcus, Corynebacterium*, *Enterobacter*, and *Propionibacterium*, where were significantly decreased in LCF samples when compared to MDL. Importantly, the MDL samples were found to be depleted of several commensals, including *Aerococcus, Lactobacillus, Jeotgalicoccus, unclassified_f__ Peptostreptococcaceae, Turicibacter, norank_f__Bacteroidales_S24-7_group, Facklamia, Prevotella_9, Lachnospiraceae_NK4A136_group, unclassified_f__ Lachnospiraceae, and Ruminococcaceae_UCG-014.* The LCF samples were enriched for *Aerococcus, Facklamia, Jeotgalicoccus, Streptococcus, Lactobacillus, norank_f__ Bacteroidales_S24-7_group, Rothia, Ruminococcaceae_UCG-014, Enterococcus, Prevotella_9, unclassified_f__Peptostreptococcaceae, and Escherichia-Shigella*. Based on these results, the LCF could modulate the skin microbiota of acne rats, resulting in a microbiota composition similar to that of CTR rats.

#### 3.7.3 Associations Between the Skin Microbiota Composition and Acne Phenotypes

**FIGURE 11:**
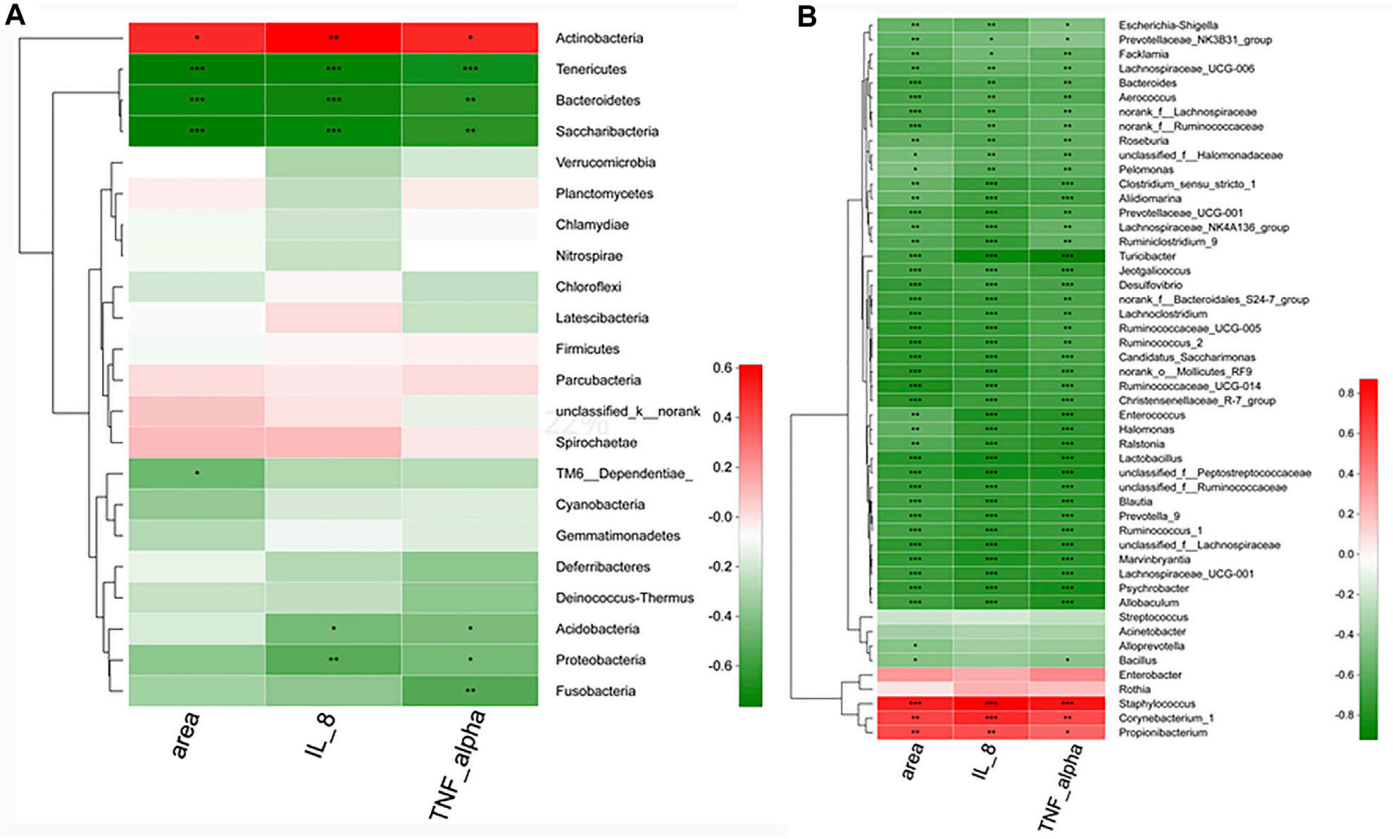
The correlation between microbes and vital pathologic parameters linked to acne. At the phylum level **(A)** and at the genius level **(B)**. *n* = 8 rats per group. *.01 < *p* < .05, **.001 < *p* ≤ .01, ***p ≤ .001.

Significant correlations were observed between the pathologic parameters with acne and the relative abundances of the skin microbial community, which were presented in the heatmap. Spearman’s correlation analysis was used to determine the correlation of each microbial level. At the phylum level (Figure 11A), *Tenericutes*, *Bacterioidetes*, and *Saccharibacteria* have exhibited a definite negative correlation with the areas of the stratified squamous epithelium, IL-8, and TNF-α concentration in serum, while *Actinobacteria* showed a positive correlation with the parameters.

At the genus level (Figure 11B), *Staphylococcus*, *Corynebacterium,* and *Propionibacterium* exhibited significant (*p* < .05) positive correlations with the pathologic parameters. However, more than 42 skin microbes, especially *Allobaculum*, *Blautia*, *Enterococcus*, *Turicibacter*, *Lactobacillus*, and *Jeotgalicoccus* has shown significant (*p* < .001) negative correlations with the areas, IL-8, and TNF-α.

#### 3.7.4 Predictions of Skin Microbiota Functions in the Control, Model, and Licorice Flavonoids Groups

**FIGURE 12:**
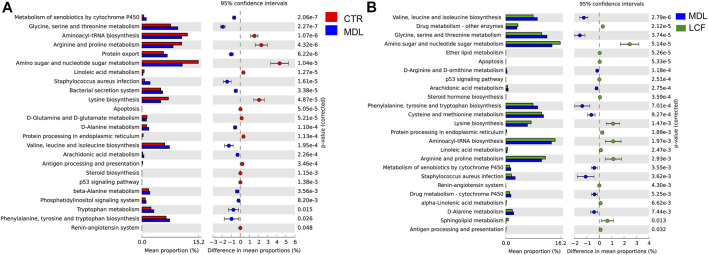
Comparisons of functional pathways in microbes from the CTR and MDL groups **(A)** and in the LCF and MDL groups **(B)**. *n* = 8 rats per group.

The mechanism by which skin microbes exert their biological effects is closely related to the function of the genes encoded in the skin microbiome. Therefore, we predicted corresponding changes in gene abundance and metabolic pathways using PICRUSt and calculated the changes in functional pathways between groups using STAMP software. In the comparison of the CTR and MDL groups, the microbiota in the latter comprised more functions involved in metabolic pathways involving glycine, serine and threonine, *staphylococcus aureus* infection, valine, leucine and isoleucine biosynthesis, and phenylalanine, tyrosine and tryptophan biosynthesis than the microbiota in the former. In contrast, the MDL group included functions involved in the aminoacyl-tRNA biosynthesis, arginine and proline metabolism, amino sugar and nucleotide sugar metabolism, and lysine biosynthesis were inhibited (Figure 12A). Regarding the comparison of the MDL and LCF groups, valine, leucine and isoleucine biosynthesis, glycine, serine and threonine metabolism, phenylalanine, tyrosine and tryptophan biosynthesis, cysteine and methionine metabolism, *staphylococcus aureus* infection, alanine metabolism, and arachidonic acid metabolism were significantly depressed (*p* < .01). However, similar to the CTR groups, the LCF group enriched more function in the amino sugar and nucleotide sugar metabolism, aminoacyl-tRNA biosynthesis, arginine and proline metabolism, and antigen processing and presentation (Figure 12B).

## 4 Discussion

### 4.1 Licorice Flavonoids Analysis

In this paper, UPLC-Orbitrap-MS was used to analyze the main components of LCF and compounds such as flavonoids, organic acids, amino acids, and alkaloids were identified. High-throughput analytical methods of UPLC-MS can cover most of the components of LCF, including LCC, Neobavaisoflavone, LCA, Diammonium glycyrrhizinate, Licoflavone A, Liguiritigenin, Sesamolin, Dimefuron, Kanzonol C, Isoliquiritigenin, Glycyrrhizic acid, Liquiritigenin, Glabrolide, Ursolic acid, Azelaic acid. Various components in LCF can exert the effect of anti-inflammatory, anti-acne, and regulate skin barrier. SooNam Park et al. (Kwon et al., 2015) prepared a transdermal formulation of hydrogel containing isoglycyrrhizin and found that it inhibited the proliferation of *C. acnes*. Besides, isoglycyrrhizin inhibits inflammatory cytokines like IL-6, IL-8, TNF-α and IL-1β in NF-κB, p38 and ERK pathways in multi cells, all of which have been reported to be closely related to acne (Wu et al., 2016; Yu et al., 2017). There is a great number of chalcones in LCF, and the special scaffold of chalcones was regarded as the key factor for their broad biological activities (Karthikeyan et al., 2015). Components of Chalcones can inhibit the cytokines including IL-6, MMP-7, MMP-8 (Karthikeyan et al., 2015), ROS, and TNF-α (Furusawa et al., 2009), which are benefited for skin barrier repairment and protection from inflammatory lesions. Besides, azelaic acid, which is used as clinical medicine in anti-acne, is also detected in LCF. LCF contains trace amounts of small molecular amino acids, which are important for maintaining skin barrier function.

### 4.2 Proteomics Analysis

Acne vulgaris is a common chronic inflammatory disease of the sebaceous follicles (Williams et al., 2012). The inflammatory response persists throughout the pathological process of acne. Cytokines like TNF-α and IL-8 have an important role in the development of inflammation of acne. Studies have shown that TNF-α and IL-8 levels in patients with acne are significantly higher than in people without acne (Abd et al., 2007). *C. acnes* binds to Toll-like receptors (TLRs) on keratinocytes, sebaceous glands and dendritic cells in the skin, activating the signal cascade of nuclear factor kappa-B (NF-κB), mitogen-activated phosphokinase (MAPK) (Kim et al., 2002). Subsequently, chemokines, including TNF-α, IL-8 and IL-6, were transcribed, which triggers inflammatory cell infiltration in acne skin (Kim et al., 2002). Stimulation of sebaceous gland cells by TNF-α and IL-8 will lead to increased lipid secretion (Hou et al., 2019). Apparently, IL-8 and TNF-α play an essential role in the pathophysiology of acne. LCF can significantly (*p* < .05) inhibit the upregulated levels of IL-8 and TNF-α in rat skin with acne, thereby inhibiting the inflammatory response. In addition, we observed the pathological improvement on rat skin with LCF treatment of acne by paraffin section of skin.

### 4.3 Plasma Metabonomics Analysis

In this paper, we carried on untargeted metabolome researches based on LC/MS to describe the metabolic profile of the skin and serum of rats with acne. Results showed that amino acid metabolism was important in rats with acne and LCF intervention. Captopril, a derivative of proline and an effective competitive inhibitor of angiotensin converting enzyme, which is responsible for the conversion of angiotensin I to angiotensin II (ATII) that is an key component of the renin-angiotensin-aldosterone system (RAAS). RAAS is involved in apoptosis, and inflammatory regulation (Lapteva et al., 2002). ATII participates in multiple stages of the inflammatory process and stimulates the expression of NF-κB (Phillips and Kagiyama, 2002). NF-κB increases the expression of pro-inflammatory factors including interleukin 1β (IL-1β), IL-6 and tumor necrosis factor alpha, three of which are essential in acne pathology (Atreya et al., 2008). We found that the expression of captopril was significantly downregulated in the skin of acne rats, and significantly upregulated after LCF intervention, suggesting that LCF may inhibit skin inflammatory response by upregulating captopril in a manner. Besides, L-Phenylalanine and L-Proline expression were decreased in the serum of acne rats and upregulated after LCF intervention. It has been reported that the expression of L-Phenylalanine and L-Proline in serum is inseparable from the inflammatory response. L-Phenylalanine was increased in patients suffering from inflammatory diseases, in which immune activity and inflammation increase the plasma (Geisler et al., 2013). L-Proline is a stress substrate in the microenvironment of inflammation (Phang et al., 2008). However, both of them acted as biomarkers in acne, their regulation mechanism is still unclear and needs further exploration. Compared with CTR, L-aspartate acid and M-tyrosine in MDL skin were significantly upregulated, aminobenzoyl-glutamate was significantly downregulated, and the three could also be used as metabolic biomarkers of skin in acne model rats.

Leucine was upregulated in MDL compared to CTR (VIP = 8.7) in the skin, and significantly downregulated after LCF intervention (VIP = 23.2, *p* < .01). Leucine is the most important biological target in the anti-acne effect of LCF. Leucine, as an activator of the mammalian target of rapamycin complex 1 (mTORC1), can prompt lipid biosynthesis and protein synthesis (Melnik, 2012). People with a high dairy protein consumption diet are more likely to suffer from acne, which was closely related to leucine signaling activated (Melnik, 2012). Leucine is a particularly potent regulator, to the extent where leucine stimulation alone is sufficient to stimulate mTORC1 signal transduction (Dodd and Tee, 2012). mTORC1 can activate the synthesis of sterol regulatory element-binding protein 1 (SREBP-1) in sebocytes, while SREBP-1 controls lipid synthesis in sebaceous glands (Bakan and Laplante, 2012). We have proved that mTORC1were activated in acne rats, then SREBP-1 had upregulated in both RNA and protein levels, but down regulated after LCF treatment through proteomics research (Ruan et al., 2020). Thus, leucine signaling can promote the development of acne by mediating the activation of SREBP-1. In this case, the leucine signal in the skin was activated in the MDL, thereby promoting lipid synthesis in the sebaceous gland. LCF can significantly inhibit the expression of leucine in the skin, inhibiting the activity of SREBP-1, reducing the biosynthesis of sebum in the sebaceous glands, and promoting the recovery of acne. Notably, in serum metabolites, leucine expression was downregulated in MDL and upregulated after LCF administration, which was completely opposite to the trend of leucine in the skin. This suggests that the leucine in the body may involve a redistribution process. Under the same feeding conditions, it means that the intake of amino acids was basically the same. After acne modeling in rats, the leucine in the blood may transfer to the local skin tissue and then accumulate, which would increase the leucine content in the skin, thereby mediating lipid synthesis in the sebaceous glands.

### 4.4 Skin Metabolomics Analysis

Lipid metabolism in the skin was another important metabolic feature of the acne and were closely related to the inflammatory response of acne. Sebum is composed largely of polar and neutral lipids including glycerolipids, free fatty acids, sterols, squalene, and GPs (Drake et al., 2008). The GPs are mainly composed of LPC, LPE, phosphatidylcholine (PC), phosphatidylethanolamine (PE). PE is one of the components that make up the cell membrane. In many diseases, PE was regarded as biomarker and regulated the inflammatory response. Lipid peroxidation can generate a variety of lipid aldehydes. PE is the major target for these lipid aldehydes, forming aldehyde-modified PE as a novel family of mediators for inflammation (Guo and Davies, 2013). The SDEMs of the skin, PE16 was significantly downregulated in MDL compared with CTR, and significantly upregulated after treatment with LCF compared with MDL. In addition, in the DEMs of the skin, the expression of PE17, PE15, LPE 20, PC14, PC18, PC20, LPC16, LPC19, LPC20 was upregulated in MDL, and LPC22 expression was downregulated. Downregulation of PE15, PE17, PE19, LPE 20, PC14, LPC19, LPC20 was observed after LCF intervention. The metabolism of phospholipids and sphingolipids were dysfunctional in acne rats, which could be restored by LCF. Although the regulation of GPs in acne is not fully understood, it is certain that LCF can maintain the lipid homeostasis of the skin by regulating the metabolism of GPs, while regulating the inflammatory response of acne.

The SDEMs of the serum, LPE 18: 1, LPC 18: 2, LPC (16: 1 (9Z)/0: 0) in MDL were significantly upregulated compared to CTR but downregulated after LCF intervention. High expression of LPC in serum causes inflammation. LPC exerts inflammatory regulation in various cell types such as T lymphocytes, monocytes, and neutrophils through different signaling pathways (Ryborg et al., 2000). Intradermal injection of LPC in healthy volunteers can also cause acute inflammation (Ryborg et al., 2000). LPC stimulated IL-8 expression and secretion in endothelial cells (Chang et al., 2017), which were related to acne lesions. Besides, LPE can also induce the increase of calcium ion concentration *via* adhesion G-protein-coupled receptor (Park et al., 2014). Epidermal keratinocyte *via* G-protein-coupled receptor causes an increase in intracellular calcium concentration, and changes in cellular calcium dynamics cause abnormal hair follicle keratinization (Katsuta et al., 2005). Increasing the concentration of LPE in the serum and skin may lead to the deterioration of acne by mediating the calcium dynamics of keratinocytes. Additionally, LPE was inhibited by LCF.

Saturated Fatty acids (SFA) and unsaturated fatty acids (UFA) are significant for the pathological changes of acne, especially for hyperkeratosis. Squalene, PA, SA, and OA can promote the secretion of IL-1 by macrophages, causing inflammation (Lovászi et al., 2017). Parsa’s work found that free fatty acids in skin lipids were significantly elevated in acne patients compared with healthy subjects (Li et al., 2017). For SDEMs in serum, the SA and OA in MDL were upregulated compared with CTR, and then downregulated in LCF, both of which regulated the pathways of SFA and UFA biosynthesis. For DEMs in the skin, linoleic acid was upregulated in MDL then downregulated in LCF. Linoleic acid induces increased lipid synthesis and secretion in sebaceous glands (Makrantonaki and Zouboulis, 2007).

In recent years, studies have shown that the activation of calcium channels in epidermal keratinocytes leads to the influx of calcium ions into cells, which was considered to be one of the causes of hyperkeratosis (Denda et al., 2003). OA can cause changes in the interstitial membrane structure and the loss of the calcium gradient in the SC. This causes the expansion of the hair follicle cavity by activating N-methyl-D-aspartic acid receptor (NMDA), resulting in excessive keratinization of the hair follicle and eventually the formation of comedo (Choi et al., 1997). Katsuta confirmed that the application of UFA (such as OA and PA) contained in sebum to the skin of hairless mice can lead to excessive keratinization of the skin (Katsuta et al., 2009). The further intervention of human keratinocytes with UFA revealed that it increased the intracellular calcium concentration of the calcium ion concentration of the cells by activating the NMDA receptor (Katsuta et al., 2009). In our study, the synthesis of SFA and UFA increased in MDL, which exacerbated the hyperkeratosis of the pilosebaceous unit. By regulating the metabolism of fatty acids, LCF inhibits the keratosis of acne to participate in the regulation of the physiological and pathological processes of acne.

Acne pathophysiology includes hyperseborrhea, inflammatory reaction, abnormal follicular keratinization and dysbacteriosis in the pilosebaceous unit (Dréno, 2017). Our results show that in the model of acne, amino acid metabolism (especially leucine), lipid metabolism (especially GPs), fatty acid metabolism (represented by OA and SA) were closely related with hyperseborrhea, inflammatory reaction, and abnormal follicular keratinization. These metabolites can also interact with each other. LCF can simultaneously regulate these three types of metabolites to treat acne.

### 4.5 Microbiota Analysis

The “microbiome” of the skin, a complex group of bacteria, viruses, and fungal organisms that inhabit all epithelial surfaces and have unique functions on the skin. The four main bacterial gates on the skin are *Actinomycetes*, *Proteobacteria*, *Absidia*, and *Bacteroides*. The composition of the skin bacterial microbes depends mainly on the characteristics of skin sites and its local chemical environment (Oh et al., 2014). Concomitant with the improved clinical index of acne rats, we observed an altered microbial composition in the LCF and MDL. Based on the PCoA, the gut microbiota structure shifted in the MDL. The LCF showed to reverse the microbial structural variations in acne rats.

The microbiome in the skin maintains a dynamic competitive state at all times. *C. acnes* inhibits the growth of *S. epidermidis., methicillin-resistant Staphylococcus aureus* (MRSA) and *streptococci. C. acnes* can create a niche that is detrimental to many skin pathogens by producing free fatty acids (FFA), including lauric acid and linoleic acid (Drake et al., 2008). What’s more, most of *S. epidermidis* can inhibit the proliferation of *C. acnes* in the skin through complex mechanisms (Wang et al., 2014). It has been reported that in the skin of acne, the abundance of S. epidermidis increases, which inhibits the proliferation of *C. acnes* (Dreno et al., 2017), reflecting a state of mutual competition in acne skin microbes. In addition, *S. epidermidis* are also opportunistic pathogens. sixty-one genes in *S. epidermidis* are involved in biofilm formation, cytotoxicity, IL-8 production, and methicillin-resistant (Méric et al., 2018). *S. epidermidis* can induce the TNF-α, IL-1 and INF-γ expression after invading into mucosa to trigger inflammation (Petropoulos et al., 2006). *Staphylococcus* and *Corynebacterium* are the two most abundant genus in the skin, and the dynamic competition between the two is important for maintaining skin homeostasis. In our research, *Staphylococcus, Corynebacterium* and *Propionibacterium* were proliferated significantly (*p* < .05) in the MDL skin when compared with the CTR. Besides, some bacteria beneficial for anti-acne in the skin, such as *Streptococcus*, *Lactococcus*, *Lactobacillus*, and *Enterococcus*, are significantly reduced. The change of the shin microbes in acne were no longer characterized by the one species over-proliferation, but by changes in the overall microbial structure in the skin, which affects the pathophysiological process of acne. Our results also shown that the associations between the skin microbiota composition and acne phenotypes, such as hyperkeratosis, IL-8 and TNF-α expression, were significantly related. It reflected that the overall change in the skin microbiota structure was related to the pathology of acne.

*Corynebacteria*, dominant members of the skin microbiota, are essential species on the regulation skin immune system. Under steady-state conditions, the impact of *Corynebacterium* is discrete and noninflammatory. However, changing the inflammatory and metabolic state of the host would stimulate *Corynebacterium,* then the skin immune system would respond. *Corynebacterium* detected on the psoriasis patients skin can increase the expression of IL-1 in the skin and increase the number and activation of γδT cells, mediating the skin inflammatory response (Cai et al., 2019). When applied to the skin of a host fed a high-fat diet, *Corynebacterium* can promote inflammation in an IL-23-dependent manner (Ridaura et al., 2018). And also, a high-fat diet is an important factor in inducing acne (Melnik and Zouboulis, 2013), *Corynebacterium* may also exert similar inflammation effects on the acne skin. In our research, over-proliferation of *Corynebacterium* was detected in the skin of acne model rats, and the inflammatory and metabolic state in acne rats were also disordered, which may activate pro-inflammatory responses under inordinate skin conditions by *Corynebacterium*.

*Propionibacterium* bacteria, which are closely related to *Corynebacterium* in the evolutionary relationship, are of great significance in the pathophysiology of acne, of which the most important is *C. acnes*. It not only affects other bacteria in the skin by regulating skin lipid synthesis but also regulates the skin’s immune response and inflammatory response. In the model group, the bacteria of the genus *Propionibacterium* were significantly increased. Excessive binding of *C. acnes* to cells leads to activation of monocyte Toll-like receptor 2 (TLR2), resulting in increased secretion of interleukin-12 (IL-12) and IL-8, while IL-12 is mononuclear. Cells are responsible for the major pro-inflammatory cytokines produced by Gram-positive organisms when invading the skin (Kim et al., 2002; Kim, 2005). *C. acnes* is an agonist of Protease-activated Receptor 2 (PAR-2) on sebocytes, and PAR-2 expression is increased in sebaceous glands of acne lesions. Lipid synthesis and SREBP-1 expression are enhanced by *C. acnes*-treated sebocytes (Lee et al., 2015). *C. acnes* can form biofilms in the hair follicle sebaceous glands, and the colony structure formed by *co-planting staphylococcus, malassezia* and *propionibacterium* in biofilm has an important influence on bacterial resistance and inflammatory ability (Jahns et al., 2013).

*Enterobacter* bacteria also increased significantly in the model group (*p* < .05) and significantly decreased after administration (*p* < .05). Enterobacteriaceae is common in skin traumatic infections (Bettoli et al., 2019). Studies have shown that *Enterobacter* cloacae can regulate inflammation and lipid metabolism in aseptic obese mice, and *Enterobacter* cloacae was also expressed in skin soft tissue infections (Yan et al., 2016). Whether its expression in the skin can regulate lipid synthesis remains to be verified.

*Streptococcus, lactococcus*, *lactobacilli* and *enterococci* in the skin are the bacteria most likely to control acne (Mottin and Suyenaga, 2018). *Enterococcus genus* belongs to the genus *Lactobacillus* and studies showed that a bacteriocin CBT-SL5 of *Enterococcus* can inhibit the expression of IL-8 induced by *C. acnes* in human keratinocytes (Mottin and Suyenaga, 2018). The study found that *Enterococcus faecalis* SL-5 can inhibit the proliferation of *C. acnes* and skin inflammation damage (Kang et al., 2009). *Streptococcus* is a type of *Streptococcus* that is a Gram-positive cocci. *In vitro* studies have shown that *S. saliva* inhibits acne by producing a bacteriocin-like inhibitory substance (Bowe et al., 2006). In addition, the K12 strain of *S. salivarius* may pass. Inhibition of the NK-κB pathway inhibits the production of the pro-inflammatory factor IL-8 in epithelial cells and keratinocytes (Cosseau et al., 2008). *Streptococcus thermophilus* can increase human keratinocytes and human skin stratum corneum ceramide levels (Di Marzio et al., 1999), and the expression of ceramide in the skin is important for inhibiting the development of acne. *Lactobacillus* belongs to the bacterium of the phylum *Lactobacillus* family. *In vitro* studies have found that *Lactobacillus paracasei* CNCM I-2116 can inhibit vasodilation, edema, mast cell degranulation and TNF-α expression caused by substance p. Skin inflammatory response accelerates the recovery of skin barrier function (Gueniche et al., 2010). Kang et al. applied human and murine *Lactobacillus reuteri* to combat the development of acne by inhibiting the proliferation of *C. acnes* and *S. epidermidis* (Kang et al., 2012). *Lactococcus* is a bacterium belonging to the genus *Streptococcus*, and *in vitro* studies have shown that bacteriocin produced by *Lactococcus* sp. HY 499 can inhibit the proliferation of *C. acnes* and *S. aureus*, and is a probiotic resistant to acne (Oh et al., 2006). In the skin group of the model group, *Balloon*, *Lactobacillus*, *Lactococcus*, *Galactococcus*, *Enterococcus*, *F. gram*, *Prevo*, *Ruminococcus*, *Rosella*, a variety of skin symbiotic bacteria such as *Psychrophila*, *Rosporium*, *Turicibacter*, *Digestive Streptococcus*, *Trichophyton*, *Bacteroides* and *Shigella Escherichia coli* were significantly reduced (*p* < .05), for acne the development is of great significance and can be used as an important biomarker for acne diseases. After administration of LCF, *Balloon*, *F. gram*, *Helicobacter*, *Streptococcus*, *Lactobacillus*, *Bacteroides*, *Rossella*, *Rumenococcus*, *Enterococcus*, *Prevot.* Compared with the model group, *Lactococcus*, *Digestive Streptococcus*, *Shigella Escherichia*, *Turicibacter*, *Psychromonium* and *Trichophyton* were significantly increased (*p* < .05). It can be seen that LCF can be restored by acne. The skin microbes caused by the mold is out of balance.

Comparison of LCF and model groups, proline, leucine and isoleucine biosynthesis, glycine, serine and threonine metabolism, phenylalanine, tyrosine and tryptophan biosynthesis, cysteine acid and methionine metabolism, *Staphylococcus aureus* infection, alanine metabolism and arachidonic acid metabolism were significantly weakened (*p* < .05). This evidence indicates that the dysregulation of the skin microbes of acne model rats may cause abnormal function of the microbes on the skin. LCF can restore the disordered skin microbes structure and also facilitate the regulation of the microbes on the skin. Recovery. In summary, the disorder of skin microbes structure in acne model rats in this study may be related to the amino acid metabolism of the body and lipid synthesis, and LCF may regulate skin protein synthesis and lipid metabolism by affecting the structure of skin microbes. This provides us with new research ideas and can be used as the next research goal.

## 5 Conclusion

In this study, non-targeted metabolomics and skin bacteriology were used to explore the anti-acne mechanism of LCF. Histopathological studies of the skin showed that LCF can effectively reduce skin inflammation and improve skin quality in rats. Non-targeted metabolomics studies have shown that LCF can play an anti-acne role by modulating metabolites closely related to sebum overflow, inflammatory response, and abnormal follicular keratoses, such as leucine, glycerol phospholipids (GPs), stearic acid (SA), and oleic acid (OA). Skin microbiota studies have shown that LCF could interfere with acne by regulating 20 biomarkers, such as *staphylococcus*, *corynebacterium*, propionate *bacillus*, *streptococcus*, *enterococcus*, *lactobacillus*, *enterococcus*, and so on. In addition, the Western blot test showed that LCF significantly inhibited the upregulation of IL-8 and TNF -α levels in the skin of acne rats, thereby inhibiting inflammation. Therefore, this study confirmed the anti-acne mechanism of LCF through regulating metabolic balance and microbial balance and protein levels. This discovery will provide theoretical guidance for the development and clinical application of this class of drugs.

## Data Availability

The data presented in the study are deposited in the NCBI repository (https://www.ncbi.nlm.nih.gov/sra/PRJNA797483), accession number PRJNA797483.
