# Supplementary material for: Explore the Anti-Acne Mechanism of Licorice Flavonoids Based on Metabonomics and Microbiome
Source: Front Pharmacol. 2022 Feb 8;13:832088. doi: 10.3389/fphar.2022.832088 (PMC8861462; doi:10.3389/fphar.2022.832088)
Supplement: Supplementary file 1 [file DataSheet1.docx]

**[Supplementary information](http://www.rsc.org/suppdata/d0/ra/d0ra06961b/d0ra06961b1.pdf)**

**Explore the Anti-acne Mechanism of Licorice Flavonoids Based on Metabonomics and Microbiome**

*Shi-Fa Ruan^1#^, Yi Hu^1#^ Wen-Feng Wu^1^, Qun-Qun Du^1^, Zhu-Xian Wang^1^, Ting-Ting Chen^1^, Qun Shen^1^, Li Liu^1^, Cui-Ping Jiang^1^, Hui Li^2^, Yankui Yi^1^, Chun-Yan Shen^1^, Hong-Xia Zhu^3*^, Qiang Liu^1*^*

^1^: School of Traditional Chinese Medicine, Southern Medical University, Guangzhou 510515, China

^2^: Department of Traditional Chinese Medicine, Guangzhou Red Cross Hospital, Jinan

University, Guangzhou,510220, China

^3^: Integrated Hospital of Traditional Chinese Medicine, Southern Medical University, Guangzhou, 510300, China

*^#^Shi-Fa Ruan and Yi Hu contributed equally to this work.*

^#^First author Email address: [schwarz_r@163.com](mailto:schwarz_r@163.com), YiHu0513@163.com.

**^*^Corresponding author:** School of Traditional Chinese Medicine, Southern Medical University, Guangzhou, 510515, China. Tel.: +86-20-6164-8264, (Qiang Liu) *Email-address*: liuqiang@smu.edu.cn

**^*^Corresponding author:** Integrated Hospital of Traditional Chinese Medicine, Southern Medical University, Guangzhou, 510315, China. Tel.: +86-20-62789408, (Hongxia Zhu) *Email-address*: gzzhx2012@163.com

Table of Contents

**1.[The Tables of main chemical constituents of licorice flavonoids S3](#_bookmark0)**

**2.The Tables of SDEMs in skin metabolites between CTR and the MDL S7**

**3.****The Tables of SDEMs in skin metabolites between LCF and the MDL S8**

**4.The Tables of Pathway analysis of skin SDEMs S9**

**5.The Tables of SDEMs between CTR and the MDL of serum S11**

**6.The Tables of SDEMs between the LCF and MDL of serum S12**

**7.The Tables of Metabolic pathways involved in SDEMs of serum S13**

**8.LCF raw data processing and analysis S15**

**9.Quality control (QC) samples preparation method S15**

**10.Data processing and analysis S15**

**1. The Tables of main chemical constituents of licorice flavonoids**

Tables. S1 Main chemical constituents in licorice flavonoids.

| **NO.** | **Compounds Name** | **Formula** | **Molecular Weight** | **RT**  **（min）** | **Relative contents(%)** |
| --- | --- | --- | --- | --- | --- |
| **1** | 4',7-Dihydroxyflavanone | C15H12O4 | 256.07393 | 3.337 | 1.03 |
| **2** | Licochalcone A | C21H22O4 | 338.15218 | 7.601 | 12.89 |
| **3** | Glycitein | C16H12O5 | 284.06892 | 4.851 | 0.32 |
| **4** | 18-β-Glycyrrhetinic acid | C30H46O4 | 470.34021 | 10.544 | 1.44 |
| **5** | Neobavaisoflavone | C20H18O4 | 322.12098 | 6.619 | 13.78 |
| **6** | Daidzein | C15H10O4 | 254.05818 | 4.447 | 1.71 |
| **7** | Retrochalcone | C16H14O4 | 270.08937 | 7.684 | 0.14 |
| **8** | Isoliquiritin | C21H22O9 | 418.12703 | 4.176 | 0.34 |
| **9** | Glycyrrhizic acid | C42H62O16 | 822.40486 | 6.517 | 0.83 |
| **10** | Diammonium glycyrrhizinate | C42H62O16 | 822.40486 | 6.115 | 5.52 |
| **11** | Osajin | C25H24O5 | 404.16249 | 12.831 | 0.11 |
| **12** | 4'-Methoxyflavone | C16H12O3 | 252.07906 | 7.602 | 0.42 |
| **13** | Liguiritigenin-7-O-β-D-  apiosyl-4'-O-β-D-glucoside | C26H30O13 | 550.16987 | 3.482 | 4.34 |
| **14** | Glabrolide | C30H44O4 | 468.32462 | 11.367 | 0.53 |
| **15** | 7-Hydroxyflavone | C15H10O3 | 238.06307 | 5.707 | 0.02 |
| **16** | Licoflavone A | C20H18O4 | 322.12141 | 6.671 | 4.34 |
| **17** | Liquiritin | C21H22O9 | 418.12727 | 0.822 | 0.16 |
| **18** | Dipotassium glycyrrhizinate | C42H60O16 | 822.40507 | 6.522 | 0.40 |
| **19** | Liquiritigenin | C15H12O4 | 256.074 | 4.737 | 0.67 |
| **20** | Isoliquiritigenin | C15H12O4 | 256.074 | 5.838 | 1.93 |
| **21** | Glabrone | C20H16O5 | 336.1 | 6.359 | 0.13 |
| **22** | Hispaglabridin B | C25H26O4 | 390.1839 | 12.103 | 0.04 |
| **23** | Licochalcone C | C21H22O4 | 338.1524 | 7.746 | 20.44 |
| **24** | Calycosin-7-O-β-D-glucoside | C22H22O10 | 446.1221 | 5.205 | 0.03 |
| **25** | 5-Hydroxy-6,7-dimethoxylflavone | C17H14O5 | 298.0841 | 5.394 | 0.06 |
| **26** | Licochalcone B | C16H14O5 | 286.0843 | 5.363 | 0.04 |
| **27** | Mulberrin | C25H26O6 | 422.1737 | 10.924 | 1.54 |
| **28** | Kuwanon G | C40H36O11 | 692.2271 | 9.199 | 0.01 |
| **29** | Formononetin | C16H12O4 | 268.07393 | 5.955 | 3.50 |
| **30** | Linolenic acid ethyl ester | C20H34O2 | 306.25597 | 12.899 | 0.03 |
| **31** | α-Eleostearic acid | C18H30O2 | 278.22483 | 9.908 | 0.03 |
| **32** | Berberine | C20H17NO4 | 335.11598 | 4.312 | 0.03 |
| **33** | Proline | C5H9NO2 | 115.06339 | 0.891 | 0.01 |
| **34** | α,α-Trehalose | C12H22O11 | 342.11665 | 0.801 | 0.11 |
| **35** | Naringenin | C15H12O5 | 272.06859 | 4.007 | 0.43 |
| **36** | Azelaic acid | C9H16O4 | 188.10472 | 4.228 | 0.02 |
| **37** | 4-Coumaric acid | C9H8O3 | 164.04741 | 3.396 | 0.004 |
| **38** | Ferulic acid | C10H10O4 | 194.05789 | 3.655 | 0.0023 |
| **39** | 5-O-Methylgenistein | C16H12O5 | 284.06871 | 6.953 | 0.07 |
| **40** | Sakuranetin | C16H14O5 | 286.0844 | 4.564 | 0.13 |
| **41** | Testosterone undecanoate | C30H48O3 | 456.36089 | 11.277 | 0.04 |
| **42** | 3-Hydroxybenzoic acid | C7H6O3 | 138.03155 | 2.357 | 0.004 |
| **43** | Daidzin | C21H20O9 | 416.11086 | 3.17 | 0.06 |
| **44** | 4-Hydroxycoumarin | C9H6O3 | 162.03161 | 2.969 | 0.004 |
| **45** | Psoralidin | C20H16O5 | 336.10045 | 8.129 | 0.55 |
| **46** | Nobiletin | C21H22O8 | 402.13191 | 6.684 | 0.03 |
| **47** | 4-O-Methylpinosylvic acid | C16H14O4 | 270.08937 | 6.558 | 0.15 |
| **48** | Anabasine | C10H14N2 | 162.11571 | 1.092 | 0.10 |
| **49** | 6-Acetylcodeine | C20H23NO4 | 341.16304 | 2.299 | 0.01 |
| **50** | Dodecanedioic acid | C12H22O4 | 230.15173 | 6.087 | 0.02 |
| **51** | Ursolic acid | C30H48O3 | 456.36145 | 12.728 | 1.38 |
| **52** | 9-Oxo-10(E),12(E)-  octadecadienoic acid | C18H30O3 | 294.21951 | 10.696 | 0.41 |
| **53** | Palmitoyl ethanolamide | C18H37NO2 | 299.28276 | 12.558 | 0.03 |
| **54** | Choline | C5H13NO | 103.09986 | 0.732 | 0.07 |
| **55** | Oleoyl ethanolamide | C20H39NO2 | 325.29823 | 12.996 | 0.06 |
| **56** | Stearamide | C18H37NO | 283.28761 | 14.543 | 0.34 |
| **57** | 8-Prenylnaringenin | C20H20O5 | 340.13133 | 8.401 | 0.43 |
| **58** | Kanzonol C | C25H28O4 | 392.19914 | 11.574 | 2.09 |
| **59** | 1-Stearoylglycerol | C21H42O4 | 358.30893 | 14.704 | 0.03 |
| **60** | 9S,13R-12-Oxophytodienoic  acid | C18H28O3 | 292.20407 | 7.212 | 0.08 |
| **61** | 7-Hydroxy-3-[4-hydroxy-3-(  3-methyl-2-buten-1-yl)phenyl]-8-(3-methyl-2-buten-1-yl)-4H- chromen-4-one | C25H26O4 | 390.18322 | 8.977 | 2.32 |
| **62** | N,N'-Diphenylguanidine | C13H13N3 | 194.08432 | 0.864 | 0.52 |
| **63** | Arachidonic acid | C20H32O2 | 322.25084 | 12.119 | 0.02 |
| **64** | 7-Demethylsuberosin | C14H14O3 | 30.09426 | 6.175 | 0.24 |
| **65** | 16-Hydroxyhexadecanoic acid | C16H32O3 | 272.23561 | 13.754 | 0.07 |
| **66** | 7-Hydroxy-2-(4- hydroxyphenyl)-4-oxo-3,4- dihydro-2H-chromen-5-yl β-D-  glucopyranoside | C21H22O10 | 434.12183 | 4.342 | 0.01 |
| **67** | Pentadecanoic acid | C15H30O2 | 242.22464 | 13.812 | 0.002 |
| **68** | Lupenone | C30H48O | 424.37087 | 13.118 | 0.009 |
| **69** | 2,2'-Methylenebis(4-methyl-6-  tert-butylphenol) | C23H32O2 | 340.24098 | 13.51 | 0.50 |
| **70** | Methyl hexadecanoate | C17H34O2 | 316.26152 | 8.852 | 0.03 |
| **71** | α-Phenylpiperidine-2-  acetamide | C13H18N2O | 218.14188 | 1.767 | 0.01 |
| **72** | Ritalinic acid | C13H17NO2 | 219.12607 | 1.813 | 0.01 |
| **73** | Corylin | C20H16O4 | 320.10512 | 7.767 | 0.11 |
| **74** | 6-Gingerol | C17H26O4 | 294.18333 | 7.267 | 0.04 |

**2. The Tables of SDEMs in skin metabolites between CTR and the MDL**

**Table. S2** SDEMs in skin metabolites between CTR and the MDL

| Metabolite name | Formula | Adduct ion name | Average Mz | Average Rt(min) | VIP | FC |
| --- | --- | --- | --- | --- | --- | --- |
| Leucine | C6H13NO2 | [M+H]+ | 132.1026 | 3.14 | 8.737 | 1.629 |
| Phosphatidylethanolamine 16 | C39H76NO8P | [M-H]- | 716.5062 | 9.04 | 1.607 | 2.01E-05* |
| Aspartate | C4H7NO4 | [M-H]- | 131.9862 | 1.69 | 1.064 | 1.655* |
| Captopril | C9H15NO3S | [M+H]+ | 218.0919 | 4.05 | 1.046 | 0.352* |
| Aminobenzoyl-glutamate | C12H14N2O5 | [M+H]+ | 267.1084 | 3.13 | 0.952 | 0.005* |
| Palmitoylcarnitine cation | C23H45NO4 | [M+H]+ | 400.3412 | 14.25 | 0.917 | 1.382 |
| M-Tyrosine | C9H11NO3 | [M-H]- | 180.0671 | 5.67 | 0.905 | 1.657* |
| 5-Fluro-2-pyrimidone | C4H3FN2O | [M+H]+ | 115.036 | 1.61 | 0.187 | 0.652 |
| Uridine | C9H12N2O6 | [M+H]+ | 245.078 | 13.74 | 0.0001 | 0.993 |

* *P* <0.01, SDEMs of skin between CTR and the MDL

**3. The Tables of SDEMs in skin metabolites between LCF and the MDL**

**Table.S3** SDEMs in skin metabolites between LCF and the MDL

| Metabolite name | Formula | Adduct ion name | Average Mz | Average Rt(min) | VIP | FC |
| --- | --- | --- | --- | --- | --- | --- |
| Leucine | C6H13NO2 | [M+H]+ | 132.1026 | 3.14 | 23.202 | 3.036* |
| Captopril | C9H15NO3S | [M+H]+ | 218.0919 | 4.05 | 1.826 | 0.323* |
| 5-Fluro-2-pyrimidone | C4H3FN2O | [M+H]+ | 115.036 | 1.61 | 1.598 | 0.232* |
| Phosphatidylethanolamine16 | C39H76NO8P | [M-H]- | 716.5062 | 9.04 | 1.160 | 4.25E-05* |
| Uridine | C9H12N2O6 | [M+H]+ | 245.078 | 13.74 | 0.952 | 0.023* |
| Palmitoylcarnitine cation | C23H45NO4 | [M+H]+ | 400.3412 | 14.25 | 0.925 | 0.846* |
| M-Tyrosine | C9H11NO3 | [M-H]- | 180.0671 | 5.67 | 0.898 | 1.346* |
| Aminobenzoyl-glutamate | C12H14N2O5 | [M+H]+ | 267.1084 | 3.13 | 0.080 | 0.087 |
| Aspartate | C4H7NO4 | [M-H]- | 131.9862 | 9.04 | 0.033 | 1.008 |

* *P*<0.01, SDEMs of skin between LCF and the MDL

**4. The Tables of Pathway analysis of skin SDEMs**

**Table. S4** Pathway analysis of skin SDEMs

| Pathway | Total | Expected | Hits | *P* | Impact |
| --- | --- | --- | --- | --- | --- |
| Cysteine and methionine metabolism | 28 | 1.0385 | 5 | 0.0030 | 0.3512 |
| Taurine and hypotaurine metabolism | 8 | 0.29672 | 2 | 0.0327 | 0.71428 |
| Nitrogen metabolism | 9 | 0.33381 | 2 | 0.0411 | 0 |
| Alanine, aspartate and glutamate metabolism（*） | 24 | 0.89016 | 3 | 0.0562 | 0.45253 |
| Arginine and proline metabolism（*） | 44 | 1.632 | 4 | 0.0765 | 0.12389 |
| Aminoacyl-tRNA biosynthesis（*#） | 67 | 2.485 | 5 | 0.0980 | 0.13793 |
| Histidine metabolism（*） | 15 | 0.55635 | 2 | 0.1042 | 0 |
| Glycine, serine and threonine metabolism | 32 | 1.1869 | 3 | 0.1122 | 0.2428 |
| Phenylalanine, tyrosine and tryptophan biosynthesis | 4 | 0.14836 | 1 | 0.1405 | 0.5 |
| D-Glutamine and D-glutamate metabolism | 5 | 0.18545 | 1 | 0.1724 | 1 |
| Linoleic acid metabolism | 5 | 0.18545 | 1 | 0.1724 | 1 |
| Biotin metabolism | 5 | 0.18545 | 1 | 0.1724 | 0.16667 |
| Sphingolipid metabolism | 21 | 0.77889 | 2 | 0.1814 | 0.01504 |
| Biosynthesis of unsaturated fatty acids | 42 | 1.5578 | 3 | 0.2017 | 0 |
| Cyanoamino acid metabolism | 6 | 0.22254 | 1 | 0.2032 | 0 |
| Glutathione metabolism | 26 | 0.96434 | 2 | 0.2506 | 0.06297 |
| Porphyrin and chlorophyll metabolism | 27 | 1.0014 | 2 | 0.2646 | 0.02205 |
| alpha-Linolenic acid metabolism | 9 | 0.33381 | 1 | 0.2891 | 1 |
| Phenylalanine metabolism | 9 | 0.33381 | 1 | 0.2891 | 0.40741 |
| Methane metabolism | 9 | 0.33381 | 1 | 0.2891 | 0.4 |
| Ascorbate and aldarate metabolism | 9 | 0.33381 | 1 | 0.2891 | 0 |
| Valine, leucine and isoleucine biosynthesis（#） | 11 | 0.40799 | 1 | 0.3412 | 0.33333 |
| Nicotinate and nicotinamide metabolism | 13 | 0.48217 | 1 | 0.3895 | 0.20833 |
| Pantothenate and CoA biosynthesis | 15 | 0.55635 | 1 | 0.4344 | 0 |
| Pyrimidine metabolism | 41 | 1.5207 | 2 | 0.4548 | 0.15318 |
| Tyrosine metabolism | 42 | 1.5578 | 2 | 0.4674 | 0.14001 |
| beta-Alanine metabolism（*） | 19 | 0.70471 | 1 | 0.5146 | 0 |
| Lysine degradation | 20 | 0.7418 | 1 | 0.5329 | 0 |
| Butanoate metabolism | 20 | 0.7418 | 1 | 0.5329 | 0 |
| Glycerophospholipid metabolism | 30 | 1.1127 | 1 | 0.6821 | 0.00093 |
| Drug metabolism-other enzymes | 30 | 1.1127 | 1 | 0.6821 | 0 |
| Purine metabolism | 68 | 2.5221 | 2 | 0.7306 | 0.00485 |
| Amino sugar and nucleotide sugar metabolism | 37 | 1.3723 | 1 | 0.7576 | 0.06921 |
| Valine, leucine and isoleucine degradation（#） | 38 | 1.4094 | 1 | 0.7668 | 0 |
| Fatty acid metabolism | 39 | 1.4465 | 1 | 0.7757 | 0.05008 |

* regulated by SDEMs in MDL; # regulated by SDEMs in LCF.

**5. The Tables of SDEMs between CTR and the MDL of serum**

**Table. S5** SDEMs between CTR and the MDL of serum

| Metabolite name | Formula | Adduct ion name | Average Mz | Average Rt(min) | VIP | FC  （MDL/CTR） | *P* |
| --- | --- | --- | --- | --- | --- | --- | --- |
| LPC 18:2 | C26H50NO7P | [M-H]- | 564.3295 | 9.61 | 43.040 | 1.95# | 0.0023 |
| Palmitic acid (PA) | C16H32O2 | [M-H]- | 255.2318 | 14.71 | 4.510 | 0.51* | 0.0217 |
| LPE 16:0 | C21H44NO7P | [M-H]- | 452.2767 | 10.06 | 3.131 | 1.88# | 0.0077 |
| Coprostanone | C27H46O | [M+H]+ | 369.3147 | 14.47 | 2.951 | 23.17# | 6.90E-07 |
| DL-beta-Hydroxybutyric acid | C4H8O3 | [M-H]- | 103.0382 | 2.47 | 2.247 | 2.55* | 0.0252 |
| Stearic acid | C18H36O2 | [M-H]- | 283.2632 | 13.22 | 2.244 | 2.05* | 0.0282 |
| LPE 18:1 | C23H46NO7P | [M-H]- | 478.2918 | 10.46 | 1.744 | 2.23* | 0.0176 |
| LPE 18:2 | C23H44NO7P | [M-H]- | 476.2768 | 9.51 | 1.699 | 1.57* | 0.0225 |
| gamma-Glutamylleucine | C11H20N2O5 | [M-H]- | 259.1292 | 3.69 | 1.262 | 2.27* | 0.0120 |
| Phosphatidylcholine lyso 16 | C24H48NO7P | [M-H]- | 524.3374 | 10.61 | 0.830 | 2.22* | 0.0265 |
| Citric acid | C6H8O7 | [M-H]- | 191.0189 | 1.95 | 0.821 | 1.94# | 0.0061 |
| Phosphatidylserine 18 | C44H82NO10P | [M-H]- | 814.5685 | 10.73 | 0.808 | 0.13* | 0.0402 |

**P<*0.05, #*P<*0.01, LPC: lysophosphatidylcholine; LPE: Lysophosphatidylethanolamine

**6. The Tables of SDEMs between the LCF and MDL of serum**

**Table. S6** SDEMs between the LCF and MDL of serum

| Metabolite name | Formula | Adduct ion name | Average Mz | | Average Rt(min) | | VIP | FC  (MDL/LCF) | *P* |
| --- | --- | --- | --- | --- | --- | --- | --- | --- | --- |
| Trans-Vaccenic acid | C18H34O2 | [M-H]- | 281.2468 | | 14.61 | | 28.437 | 2.96* | 0.0112 |
| Cholic acid | C24H40O5 | [M-H]- | 407.2795 | | 7.94 | | 13.559 | 2.96# | 0.0041 |
| OA | C18H34O2 | [M-H]- | 281.2462 | | 14.74 | | 5.889 | 2.25# | 0.0094 |
| Octadecanoic acid | C18H36O2 | [M-H]- | 283.2632 | | 13.22 | | 2.962 | 3.66# | 0.0034 |
| Thymidine | C10H14N2O5 | [M-H]- | 241.0536 | | 7.95 | | 2.198 | 2.03* | 0.0225 |
| LPE18:1 | C23H46NO7P | [M-H]- | 478.2918 | | 10.46 | | 1.594 | 2.18* | 0.0145 |
| Coprostanone | C27H46O | [M+H]+ | 369.3147 | | 14.47 | | 1.459 | 2.04* | 0.0215 |
| L-Phenylalanine | C9H11NO2 | [M+H]+ | 166.1002 | | 5.00 | | 1.306 | 0.53* | 0.0441 |
| L-Arginine | C6H14N4O2 | [M+H]+ | 175.1306 | | 1.14 | | 1.278 | 0.54* | 0.0215 |
| L-Proline | C5H9NO2 | [M+H]+ | 116.0935 | | 1.51 | | 0.871 | 0.45# | 0.0037 |
| Phosphatidylcholine lyso 16 | C24H48NO7P | [M-H]- | | 524.3374 | | 10.61 | 0.841 | 2.49* | 0.0288 |

**P<*0.05, #*P<*0.01, LPC: lysophosphatidylcholine; LPE: Lysophosphatidylethanolamine

**7. The Tables of Metabolic pathways involved in SDEMs of serum**

**Table. S7** Metabolic pathways involved in SDEMs of serum

| Pathways | Metabolite | FC(MDL/CTR) | FC(MDL/LCF) |
| --- | --- | --- | --- |
| Phenylalanine, tyrosine and tryptophan biosynthesis | L-Phenylalanine | 0.64* | 0.53# |
| Phenylalanine metabolism | L-Phenylalanine | 0.64* | 0.53# |
| Biosynthesis of unsaturated fatty acids | Stearic acid | 2.05# | 3.66# |
|  | OA | 1.59* | 2.25# |
|  | PA | 0.76* | 1.65* |
| Aminoacyl-tRNA biosynthesis | L-Leucine | 0.70* | 0.68* |
|  | L-Phenylalanine | 0.64* | 0.53# |
|  | L-Proline | 0.60* | 0.45# |
|  | L-Arginine | 1.71 | 0.54# |
| Pyrimidine metabolism | Thymidine | 1.44* | 2.03# |
| Citrate cycle (TCA cycle) | Citric acid | 1.94# | 0.80 |
|  | Succinic acid | 8.76* | 3.26* |
| Fatty acid biosynthesis | Stearic acid | 2.05# | 3.66# |
|  | OA | 1.59* | 2.25# |
|  | PA | 0.76* | 1.65* |
| Valine, leucine and isoleucine biosynthesis | L-Leucine | 0.70* | 0.68* |
| Arginine and proline metabolism | L-Proline | 0.60* | 0.45# |
|  | L-Arginine | 1.71 | 0.54# |
| Glyoxylate and dicarboxylate metabolism | Citric acid | 1.94# | 0.80 |
| Propanoate metabolism | Succinic acid | 8.76* | 3.26* |
| Butanoate metabolism | Succinic acid | 8.76* | 3.26* |
|  | D-beta-Hydroxybutyric acid | 2.55# | 2.02* |
| Alanine, aspartate and glutamate metabolism | Succinic acid | 8.76* | 3.26* |
| Fatty acid elongation in mitochondria | PA | 0.76* | 1.65* |
| Valine, leucine and isoleucine degradation | L-Leucine | 0.70* | 0.68* |
| Fatty acid metabolism | PA | 0.76* | 1.65* |

**P<*0.05 or VIP>0.08, #VIP>0.8 and *P<*0.05

**8. LCF raw data processing and analysis**

The raw data of the LC-MS was imported into the SraceFinder and Compound Discoverer (Thermo Scientific) software for preprocessing, including peak extraction, denoising, deconvolution, peak alignment, and exporting a three-dimensional data matrix (raw data matrix) in CSV format. This three-dimensional matrix includes information such as sample information, retention time, mass-to-nuclear ratio, and mass spectral response intensity (peak area). The ion peaks with more than half of the missing values in the group of the original data matrix were deleted. The extracted peak information was searched in three databases of Chemspider、mzCloud、mzVault to obtain information on the molecular type and molecular structure of the compounds. Cooounds that meet mzCloud values ≥ 80 and mzVault values ≥ 80 were considered as main components in LCF.

**9 Quality control (QC) samples preparation method**

The QC samples was subjected to the analysis to verify the stability of the system. An aliquot of 10 µL of each skin or serum sample was pooled to provide a quality control (QC) sample and kept at -80℃ until use. The QC sample was subjected to 5 replicate injections before the start of the analysis to verify the stability of the system. Every 3 samples tested, one was inserted to monitor and evaluate the stability of the system and the reliability of the experimental data.

**10 Data processing and analysis**

The raw data of the LC-MS was imported into the MS-DIAL 2.76 software for preprocessing, including peak extraction, denoising, deconvolution, peak alignment, and exporting a three-dimensional data matrix (raw data matrix) in CSV format. This three-dimensional matrix includes information such as sample information, retention time, mass-to-nuclear ratio, and mass spectral response intensity (peak area). The ion peaks with more than half of the missing values in the group of the original data matrix were deleted. The extracted peak information was searched in three databases of MassBank, Respect, and GNPS (14,951 records) to obtain information on the molecular type and molecular structure of the metabolite.
